# Psychological interventions for the prevention of depression relapse: systematic review and network meta-analysis

**DOI:** 10.1038/s41398-023-02604-1

**Published:** 2023-09-28

**Authors:** Yurong Zhou, Defeng Zhao, Xiaotong Zhu, Lu Liu, Ming Meng, Xiaojun Shao, Xueyan Zhu, Jing Xiang, Jiali He, Yimeng Zhao, Yuman Yuan, Rui Gao, Lin Jiang, Gang Zhu

**Affiliations:** 1 Department of Psychiatry, The First Affiliated Hospital of China Medical University, Shenyang, 110001 China; 2 Clinical Medicine (5 + 3), China Medical University, Shenyang, 110122 China; 3 Department of Psychiatry, The Fourth Affiliated Hospital of China Medical University, Shenyang, 110001 China; 4 Shenyang Mental Health Center, Shenyang, 110168 China

**Keywords:** Depression, Bipolar disorder

## Abstract

Depression is highly prevalent and easily relapses. Psychological interventions are effective for the prevention of depression relapse. This systematic review and network meta-analysis aimed to compare the efficacy at the same follow-up time points of psychological interventions in depression. We searched PubMed, Embase, and PsycINFO via OVID, and the Cochrane Library published up to December 12, 2021, and PubMed up to July 1, 2022. The primary outcome was depression relapse, considering the same time points that were extracted on survival curves or relapse curves. The study protocol was registered with PROSPERO, CRD42022343327. A total of 2,871 patients were included from 25 RCTs. Mindfulness-based cognitive therapy (MBCT) was significantly better than placebo at the 3 months, the 6 months, and the 9 months at follow-up. Cognitive behavioral therapy (CBT) was significantly better than treatment as usual at the 3 months, the 9 months, the 12 months, and the 15 months at follow-up. CBT was significantly better than placebo at the 21 months and the 24 months at follow-up. Behavioral activation therapy was significantly better than placebo at the 21 months and the 24 months at follow-up. Interpersonal psychotherapy was significantly better than placebo at the 24-month follow-up. All psychological interventions included in the study were significantly better than supportive counseling most of the time. The results were robust in various sensitivity and subgroup analyses. In conclusion, MBCT had a continuous effect in preventing relapse of depression. CBT had the longest but not continuous effect in preventing relapse of depression. The effects of behavioral activation therapy and interpersonal therapy for the prevention of depression appeared late. All psychological interventions included in the study were more effective than supportive counseling. More evidence is needed from large comparative trials that provide long-term follow-up data.

## Introduction

Depression is a kind of mental disease with symptoms of low mood and lack of experience, interest, or pleasure, and it has the highest global burden in terms of disability years lost [1]. The prevalence of depression is high, with almost one in ten patients on average experiencing depressive symptoms [2, 3], increasing the risk of self-harm and suicide [4–6]. Depression is known to relapse at a high rate; evidence indicates that a total estimated 85% of those who recovered from depression will relapse [7]. About 50% of patients experience a relapse or recurrence after recovering from the first episode of depression [8], and patients with five previous episodes of depression are more than twice as likely to relapse compared to those with one-lifetime episodes [9]. Problems concentrating and remembering continue after the depressive episode has subsided and worsens with repeated episodes [10].

Many factors influence relapse, with the more highly recognized causes being residual depressive symptoms at the end of acute treatment and a history of previous relapse [11–13]. Increasing numbers of studies have identified lack of social support, age at first presentation, comorbid mental disorders, family history of depression, neurotic personality, and major life events [14–18] as possible risk factors for relapse of depression. Recurrent episodes of depression can have a significant impact on a person’s life. Most patients who experience a relapse of depression feel limited in their productivity and social activities.

Evidence indicates that pharmacotherapy, psychotherapy, and the combination of the two interventions can prevent relapse of depression [19–21]. Psychotherapies such as cognitive behavioral therapy (CBT), mindfulness-based cognitive therapy (MBCT), interpersonal psychotherapy, etc., can effectively prevent the recurrence of depression in patients in remission [22–26]. MBCT was more effective than treatment as usual (TAU) for the prevention of relapse of depression and has an advantage over TAU and placebo for the time to relapse of depression [27]. In addition, for patients with three or more previous episodes, MBCT [24] is more effective in preventing relapse. Pharmacotherapy, whether continued or maintained, is a robust treatment for preventing relapse of depression [28].

Previous meta-analyses have analyzed the effectiveness of various interventions to prevent the recurrence of depression. The beneficial effects of CBT alone [29] in reducing the relapse of depression were equal to the beneficial effects of antidepressant medication alone. For short-term follow-up (12 months) [23], CBT was more efficacious than control in preventing depression relapse, however, MBCT and maintenance antidepressant (ADM) medication were not significantly different from each other after 2 years. Zhou et al. [30] showed that various psychological interventions were effective at preventing relapse, but they did not evaluate the effect of psychotherapies on preventing depression at different follow-up durations. Therefore, we conducted a systematic review and network meta-analysis to explore the long-term effects of different psychological interventions to prevent relapse of depression by extracting data from different follow-up time points.

## Methods

### Search strategy and selection

In this systematic review and network meta-analysis, we searched PubMed, Embase, the Cochrane Library databases, and PsycINFO via OVID from the date of their inception to December 12, 2021, and PubMed up to June 30, 2022, for studies that compared psychological interventions for depression relapse prevention. The search term included terms related to depression and depression-like disorders and a great variety of terms related to psychological interventions. The search strategy is shown in Supplementary Table 2. In addition, we examined references from the most current systematic reviews and meta-analyses [27, 30, 31]. Two researchers individually assessed all abstracts, then read full texts and chose relevant randomized controlled trials (RCTs). Discussion and communication with a third author were used to work out any differences before a decision was made. Protocol changes are detailed and presented in Appendix 1. We used common clinical psychotherapy as defined by Cuijpers et al. [31] (detailed definitions in Supplementary Table 4), and all interventions included in the study were classified as CBT, behavioral active therapy, MBCT, interpersonal therapy, supportive counseling, and others, regardless of the delivery format (e.g., individual or group) or treatment medium (face-to-face or online). There were also three groups (treatment as usual, placebo, and antidepression) included for comparison, whose detailed definitions were presented in Supplementary Table 4.

The inclusion criteria were: an RCT in which one arm included a psychological intervention combined with ADM or not; adults (≥18 years) with a diagnosis of depression but not at the acute phase at the time of randomization; survival curves or relapse curves were available for at least 6 months. We excluded studies in which the patient sample included some proportion of patients with bipolar disorder, those in which the participants were elderly individuals only, and those in which participants were male or female only.

### Data synthesis and data extraction

We extracted information about publication year; age; sex; total number of participants randomly assigned; number of episodes; follow-up duration; criteria of diagnosis and relapse; all psychological interventions such as CBT, MBCT, interpersonal therapy, behavioral active therapy or supportive counseling, and control interventions including antidepressants, placebo or treatment as usual (definitions in Supplementary Table 4). We used the Cochrane Collaboration’s risk of bias tool [32], which assesses potential sources of bias in randomized controlled trials. Two independent researchers extracted the outcome data and assessed the risk of bias, with differences of opinion resolved by consensus after discussion with another author.

The primary outcome was relapse. We extracted data for relapse at eight different time points separately (3 months, 6 months, 9 months, 12 months, 15 months, 18 months, 21 months, and 24 months). We used GetData Graph Digitizer software (version 2.26.0.20) [33] to extract data from relapse curves or survival curves.

### Statistical analysis

We performed random-effects network meta-analysis in the frequentist framework (mvmeta and network package) in Stata software (version 15.0) [34]. We used a strict intention-to-treat (ITT) approach to calculate odds ratios (ORs) for binary outcomes and standardized mean differences (SMDs) for continuous outcomes. Both results have been presented with 95% confidence intervals (CIs). Using the method described by Tierney et al. [35], the OR with 95% CI has been estimated using survival plots. The surface under the cumulative ranking curve (SUCRA) and the probabilities of being the best were estimated to rank the probabilities of each treatment. Better efficacy means a better SUCRA value. A matrix analysis was carried out to test whether the difference in the effectiveness of each pair of psychological interventions with a different SUCRA reached significance. We analyzed the distribution of clinical and methodological characteristics (such as age, sex, sample size, and year of publication) that might serve as impact modifiers across treatment comparisons to evaluate transitivity. Two methods were used to assess network consistency, the extent to which the included studies are statistically and substantively comparable. Using a loop-specific approach, we estimated the inconsistency factor in each loop as the absolute difference between the direct and indirect estimates and truncated the CIs to 0. We used a Z-test to decide whether the inconsistency was significant [36] (i.e., the lower limit of the 95% CI of the inconsistency factor touches 0). Furthermore, the estimated direct and indirect treatment effects and their difference were reported using the side-split method [37], with consistency inferred on the basis of the *p*-values of the difference. We undertook five prespecified sensitivity analyses for primary outcomes by limiting the analysis to studies reporting the narrowly defined diagnosis of major depression, limiting to studies reporting the narrowly defined relapse of major depression, limiting to studies in which the population was in remission at the time of grouping, and limiting to studies in which the patients were not suffering the first episode of depression, excluding arms that included placebo. To evaluate publication bias, we employed comparison-adjusted funnel plots. We evaluated the confidence in the relative treatment effect estimated in the network meta-analysis for the primary outcome using the confidence in network meta-analysis (CINeMA) [38], implemented in the web application CINeMA [39]. For network meta-analysis, we adhered to the PRISMA guidelines. The systematic review and network meta-analysis were already registered in PROSPERO (CRD42022343327).

## Results

### Study characteristics

**Fig. 1:**
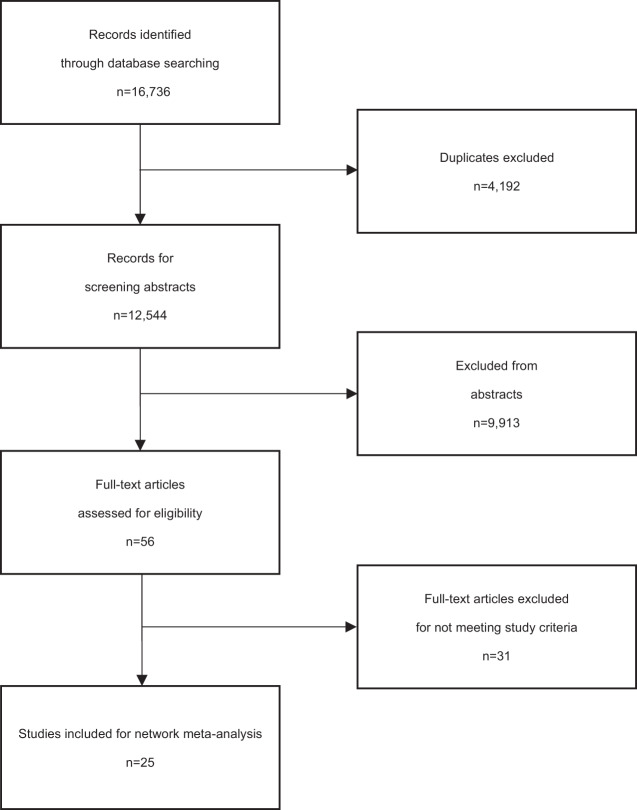
Flow chat of study selection.

After examining 12,544 abstracts (4192 after removal of duplicates), 56 full-text studies were retrieved for further scrutiny. From these studies, 25 randomized controlled trials with 2871 patients were included in the network meta-analysis (Fig. 1). The aggregated characteristics of the 25 included studies are presented in Supplementary Table 3. Searches identified a total of 2871 publications, with sample sizes ranging from 34 to 424. Study durations ranged from 7 months to 144 months. Thirteen of the 25 studies employed a follow-up period of at least 24 months. All studies had a follow-up duration of at least 12 months except Morokuma 2013 [40] and Perlis 2002 [41]. Twenty-three studies included diagnostic criteria for people with depression from the Diagnostic and Statistical Manual of Mental Disorders Fourth Edition (DSM-IV) or DSM-III. For the majority of studies, the diagnostic criteria for depressive relapse were based on DSM-III or DSM-IV diagnosis of MDD during follow-up.

Details of the risk of bias assessment for the included RCTs are presented in Supplementary Fig. 1 in the Supplement. All studies were judged to have low or unclear risk of bias for the domains random sequence generation, allocation concealment, blinding of participants and personnel, or selective reporting. Participant blinding is not possible with group-delivered psychotherapies, therefore, all studies were judged to have a high risk of bias for the blinding of participants and personnel. Three studies were judged to have a high risk of bias for the other biases because participants preferred psychotherapy interventions. Two studies were judged to have a high risk of bias for the incomplete outcome data domain, as only data from participants who completed the trial were included in the analysis.

### Network meta-analysis

**Fig. 2:**
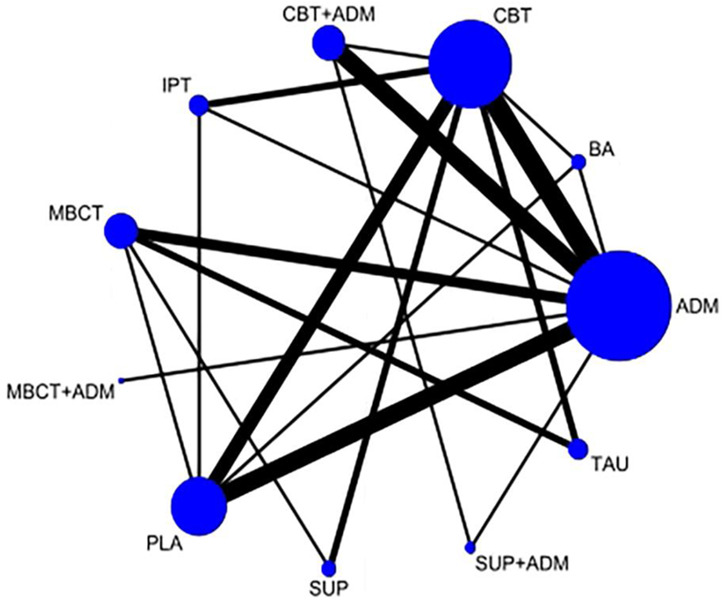
Network plot of the primary outcome of relapse at 9 months. The lines link treatments with direct comparisons in trials. The thickness of the lines corresponds to the number of trials evaluating the comparison. The size of the nodes corresponds to the number of trials investigating the intervention. ADM antidepressant, BA behavioral active therapy, CBT cognitive behavioral therapy, CBT + ADM cognitive behavioral therapy combined with antidepressant, IPT interpersonal therapy, MBCT mindfulness-based cognitive therapy, MBCT + ADM mindfulness-based cognitive therapy combined with antidepressant, PLA placebo, SUP supportive counseling, SUP + ADM supportive counseling combined with antidepressant, TAU treatment as usual.

The network plot at the 9-month follow-up is shown in Fig. 2. It indicated that CBT was the best therapy of those examined and was connected to the nodes of all other monotherapy psychological interventions (except MBCT). Five types of psychotherapy (CBT, MBCT, behavioral activation therapy, interpersonal psychotherapy, and supportive counseling) and one combined therapy (CBT plus ADM) were included in all durations of follow-up. The follow-up duration of all kinds of combined therapies was no longer than 18 months, except for CBT plus ADM and supportive counseling plus ADM (Supplementary Fig. 2).

**Fig. 3:**
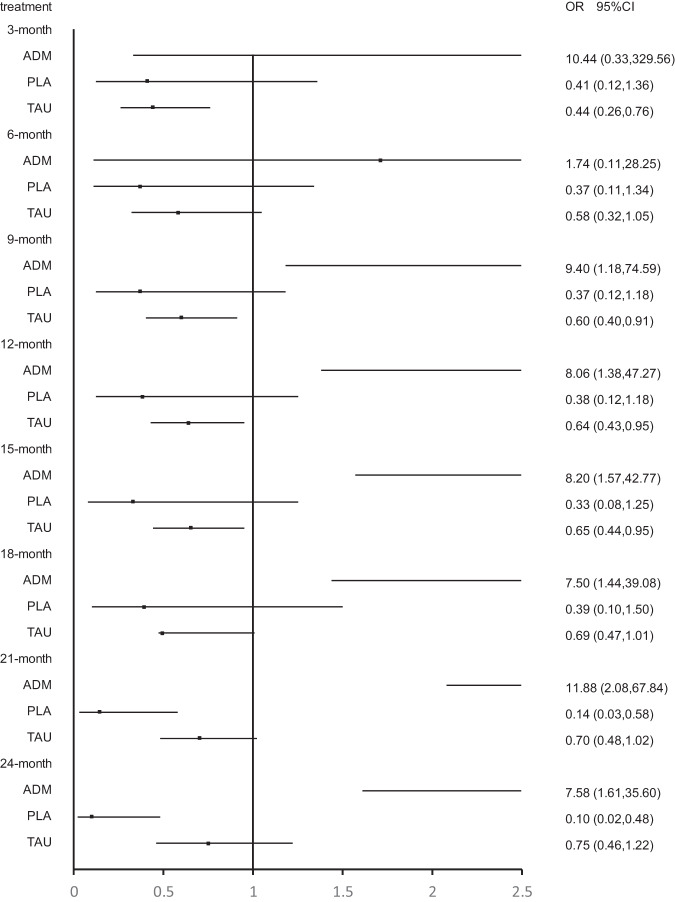
Forest plots of CBT versus antidepressant, treatment as usual, and placebo for the primary outcome of relapse at 3 months, 6 months, 9 months, 12 months, 15 months, 18 months, 21 months, and 24 months. The reference treatment is CBT. ORs less than 1 are in favor of the CBT. OR odds ratio.

**Fig. 4:**
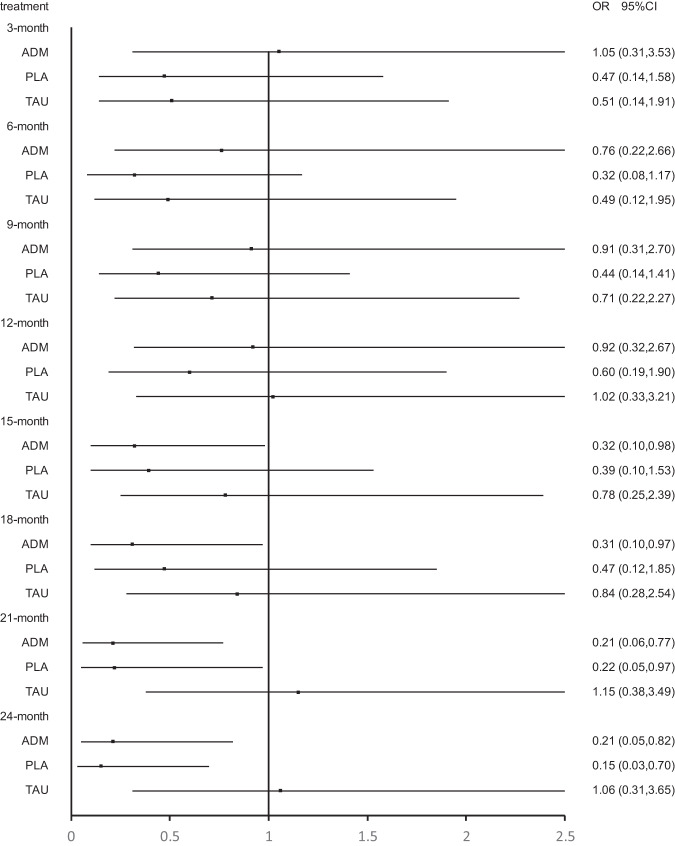
Forest plots of BA versus antidepressant, treatment as usual, and placebo for the primary outcome of relapse at 3 months, 6 months, 9 months, 12 months, 15 months, 18 months, 21 months, and 24 months. The reference treatment is BA. ORs less than 1 are in favor of the BA. OR odds ratio.

**Fig. 5:**
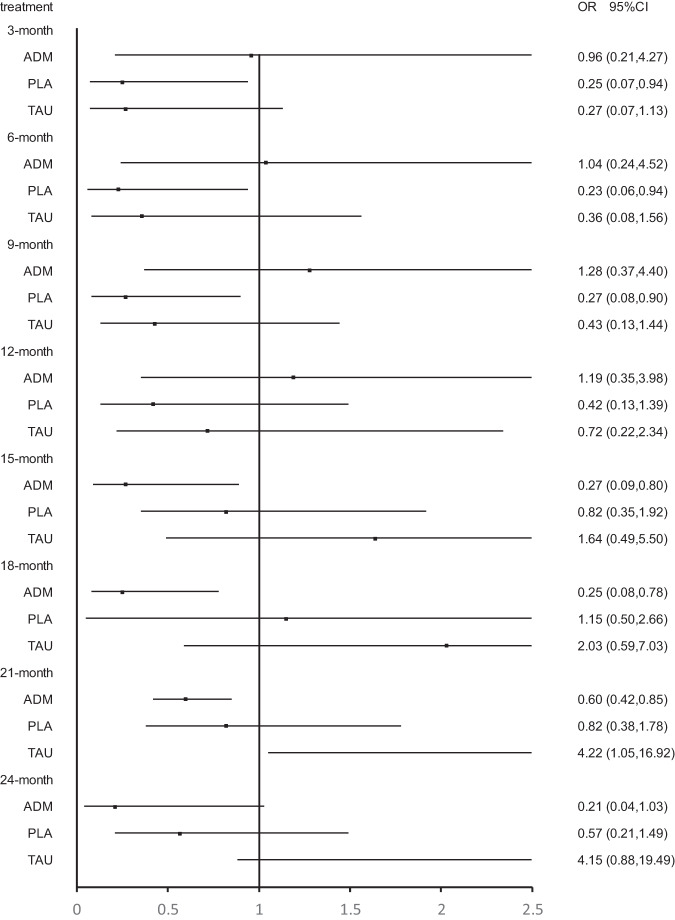
Forest plots of MBCT versus antidepressant, treatment as usual, and placebo for the primary outcome of relapse at 3 months, 6 months, 9 months, 12 months, 15 months, 18 months, 21 months, and 24 months. The reference treatment is MBCT. ORs less than 1 are in favor of the MBCT. OR odds ratio.

**Fig. 6:**
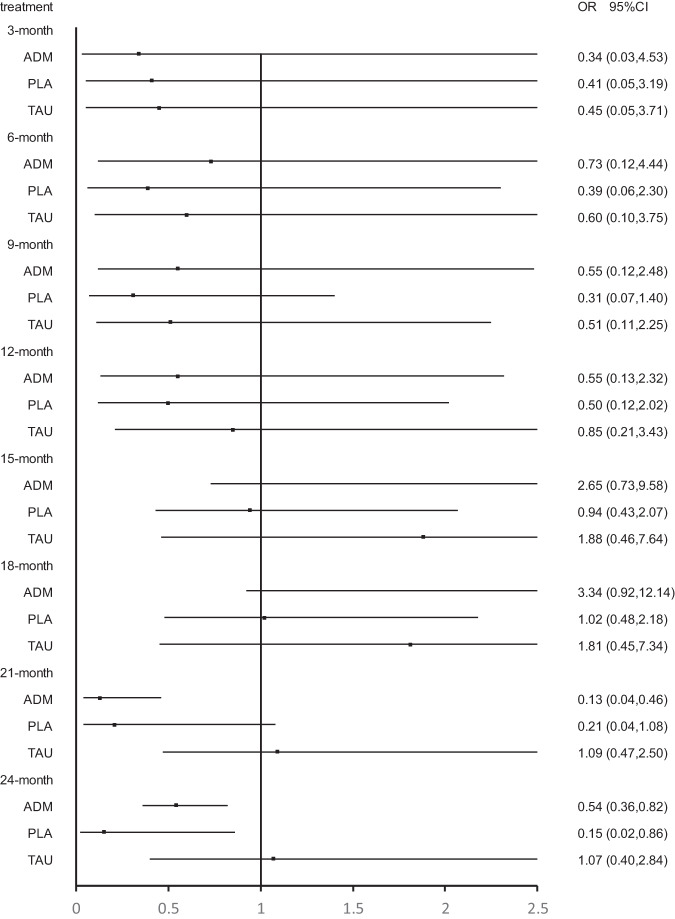
Forest plots of IPT versus antidepressant, treatment as usual, and placebo for the primary outcome of relapse at 3 months, 6 months, 9 months, 12 months, 15 months, 18 months, 21 months, and 24 months. The reference treatment is IPT. ORs less than 1 are in favor of the IPT. OR odds ratio.

Twenty-three studies were included at the 3 months follow-up. MBCT (OR, 4.33; 95% CI, 1.06–17.76) and supportive counseling plus ADM (OR, 37.80; 95% CI, 2.66–536.24) showed a significant advantage over placebo. In addition, CBT (OR, 5.45; 95% CI, 1.08–27.49), MBCT (OR, 8.87; 95% CI, 1.08–73.16), MCBT plus ADM (OR, 26.56; 95% CI, 1.31–536.52) and supportive counseling plus ADM (OR,75.99; 95% CI, 2.00–2892.65) showed significant advantage over supportive counseling. However, no psychological intervention showed a significant advantage over ADM (Figs. 3–6; Supplementary Fig. 3 and Supplementary Table 5).

Twenty-three studies were included at the 6 months follow-up. MBCT (OR, 4.33; 95% CI, 1.06–17.76) and supportive counseling plus ADM (OR, 37.80; 95% CI, 2.66–536.24) showed a significant advantage over placebo. CBT (OR, 8.00; 95% CI, 1.52–42.23), behavioral activation therapy (OR, 9.50; 95% CI, 1.19–76.09), MBCT (OR, 12.98; 95% CI, 1.52–110.60) and supportive counseling plus ADM (OR, 113.38; 95% CI, 5.09–2525.53) were also significantly better than supportive counseling. No significant difference was found between psychological interventions and ADM (Figs. 3–6; Supplementary Fig. 3 and Supplementary Table 5).

Twenty-four studies were included at the 9-month follow-up. MBCT (OR, 3.71; 95% CI, 1.11–12.39) and supportive counseling (OR, 32.45; 95% CI, 2.58–407.80) showed a significant advantage over placebo. All psychological interventions showed a significant advantage over supportive counseling. However, network meta-analysis found that ADM was significantly better than CBT (OR, 0.11; 95% CI, 0.01–0.84) (Figs. 3–6; Supplementary Fig. 3 and Supplementary Table 5).

Twenty-three studies were included at 12 months follow-up. No significant difference was found between placebo and other interventions. CBT plus ADM (OR, 7.14; 95% CI; 1.03-49.48), CBT (OR, 4.50, 95% CI; 2.03-10.00) and MBCT (OR, 4.01, 95% CI; 1.02-15.80) showed significant advantage over supportive counseling. However, no interventions except CBT plus placebo showed a significant advantage over placebo. Meanwhile, all psychological interventions showed no significant difference from ADM except CBT (OR, 0.12, 95% CI; 0.02-0.73) (Figs. 3–6; eFigure 3 and eTable 5 in the Supplement).

Twenty studies were included at the 15-month follow-up. No significant difference was found between placebo and other interventions. CBT (OR, 3.81; 95% CI, 1.86–7.80) showed a significant advantage over supportive counseling. Behavioral activation therapy (OR, 3.12; 95% CI, 1.02–9.55), MBCT (OR, 3.75; 95% CI, 1.25–11.24) and MBCT plus ADM (OR, 1.90; 95% CI, 1.32–2.73) showed significant advantage over ADM; however, ADM was found to be significantly better than CBT (OR, 0.12; 95% CI, 0.02–0.64). No psychological intervention showed a significant advantage over supportive counseling except CBT (OR, 3.81; 95% CI, 1.86–7.80) (Figs. 3–6; Supplementary Fig. 3 and Supplementary Table 5).

Seventeen studies were included at the 18 months follow-up. No psychological intervention showed a significant advantage over placebo; however, placebo showed a significant advantage over ADM (OR, 0.54; 95% CI, 0.38–0.77). Meanwhile, no psychological intervention showed a significant advantage over supportive counseling except CBT (OR, 3.59; 95% CI, 1.78–7.27). Behavioral activation therapy (OR, 3.23; 95% CI, 1.03–10.11) and MBCT (OR, 3.92; 95% CI, 1.28–12.01) showed a significant advantage over ADM, but ADM was significantly better than CBT (OR, 0.13; 95% CI, 0.03–0.69) (Figs. 3–6; Supplementary Fig. 3 and Supplementary Table 5).

Fourteen studies were included at the 21-month follow-up. CBT (OR, 7.32; 95% CI, 1.72–31.04) and behavioral activation therapy (OR, 4.48; 95% CI, 1.04–19.36) showed a significant advantage over placebo. Behavioral activation therapy (OR, 4.80; 95% CI, 1.30–17.64), MBCT (OR, 1.71; 95% CI, 1.22–2.42), and interpersonal therapy (OR, 7.84; 95% CI, 2.18–28.22) showed significant advantage over ADM; however, ADM was found to be significantly better than CBT (OR, 0.08; 95% CI, 0.01–0.48) and CBT plus ADM (OR, 0.10; 95% CI, 0.02–0.41) (Figs. 3–6; Supplementary Fig. 3 and Supplementary Table 5).

Thirteen studies were included at the 24-month follow-up. Behavioral activation therapy, interpersonal therapy, and CBT plus ADM showed a significant advantage over ADM, however, ADM was found to be significantly better than CBT. Only CBT (OR, 9.84; 95% CI, 1.79–54.15) and behavioral activation therapy (OR, 6.89; 95% CI, 1.23–38.62) showed a significant advantage over placebo. No psychological intervention showed a significant advantage over supportive counseling except CBT (OR, 3.21; 95% CI, 1.35–7.62) (Figs. 3–6; Supplementary Fig. 3 and Supplementary Table 5).

The funnel plot indicated that there was no publication bias (Supplementary Fig. 4). The transitivity assumption was not violated for any of the potential effect modifiers that were analyzed (Supplementary Fig. 5). Tests of local incoherence at 9-month follow-up time points showed that the percentages for inconsistent loops were within the expected ranges based on the empirical data (three of 15 loops; Supplementary Table 6). The test of incoherence from the node-splitting model showed significant differences between some comparisons in efficacy and acceptability (Supplementary Table 6). The sensitivity analyses did not materially affect the relative treatment effects (Supplementary Tables 7–11). According to CINeMA, confidence in estimates was low to very low for most primary outcomes at 9-month follow-up, meaning that further research is very likely to affect our confidence in the estimate of effect and is likely to change the estimate (eCINeMA in the Supplement).

## Discussion

To our knowledge, we have undertaken the first network meta-analysis investigating psychological interventions for the prevention of depression relapse at the same follow-up time point. We analyzed psychological interventions reported in 25 randomized controlled trials with patients. MBCT was better than placebo at the 3 months, the 6 months, and the 9 months at follow-up, was better than ADM at the 15 months, the 18 months, and the 21 months at follow-up. CBT was better than treatment as usual at the 3 months, the 9 months, the 12 months, and the 15 months at follow-up. CBT was significantly better than placebo at the 21 months and the 24 months at follow-up, CBT was less effective than ADM at the 9 months, the 12 months, the 15 months, the 18 months, the 21 months, and the 24 months at follow-up. Behavioral activation therapy was better than ADM at the 15 months, the 18 months, the 21 months, and the 24 months at follow-up, was better than placebo at the 21 months and the 24 months at follow-up. Interpersonal psychotherapy was better than placebo at the 24-month follow-up. All psychological interventions included in the study were significantly better than supportive counseling most of the time.

We found that MBCT demonstrated a sustained and stable effect over placebo in preventing relapse of depression early in follow-up (up to 9-month follow-up). Previous meta-analysis has found the same result [24, 27, 42–44]. Most randomized trials of manualized MBCT were followed up for less than 15 months, and the only two papers included in our study that reported more than 15 months of follow-up showed no significant difference from the control. More studies are needed to confirm the efficacy of long-term follow-up of MBCT.

CBT is more effective than other psychotherapies for preventing relapse of depression, but the effect is not stable. Prior reviews suggest that CBT may reduce relapse of depression [45]. For short-term follow-up (a 12-week period), no significant advantage was found for CBT over antidepressants [46]. However, other studies have found that CBT was significantly better than antidepressants [47–49]. At 1-year follow-up, some studies demonstrated no significant advantage for CBT over other interventions (e.g., continuation of medication and treatment as usual) [50, 51], but other studies found that CBT showed a significant advance over antidepressants [52]. In addition, among patients with five or more previous episodes of depression, CBT was significantly better than treatment as usual [50] and manualized psychoeducation [53]. Antidepressants were found to be significantly better than CBT, it was different from the previous review [30, 45]. The finding that antidepressants were significantly better than CBT differed from previous reviews [54] may explain this result.

The effects of behavioral activation therapy and interpersonal psychotherapy appeared later. This result was similar to those of previous reports [25, 55, 56]. Furthermore, those who continued their study for at least 2 years noted that the majority of recurrences (79%) occurred during the first year of maintenance treatment [55, 57]. It was found shortly after initial recovery is a high-risk time period for relapse [58]. This may explain the late onset of interpersonal psychotherapy and behavioral activation.

Previously, one network meta-analysis by Zhou et al. [30] had been performed to demonstrate the effect of psychotherapy in preventing relapse of depression. They found that most psychotherapeutic interventions, except interpersonal psychotherapy, were significantly better than ADM. We found similar results at an 18-month follow-up and that all psychotherapeutic interventions except MBCT and supportive counseling showed a significant advantage over ADM at a 24-month follow-up. Our results for MBCT were similar to those of a network meta-analysis by McCartney et al. [27] that showed that MBCT was not statistically different from m-ADM, active control condition, and cognitive therapy. The meta-analysis by Zhang et al. [23] showed that MBCT, compared with m-ADM, did not show a significant relapse prevention effect at 24-month follow-up, but for this comparison, only one study was included. This inconsistency might have occurred because we used a different outcome measure and included studies with survival curves or relapse curves. Therefore, the evidence we included was insufficient, and relevant trials need to be repeated to confirm or overturn our findings.

At the 24-month time point, only one psychological intervention (CBT plus antidepressants) was included that showed a significant effect over antidepressants. This was similar to the findings of meta-analyses by Breedvelt et al. [20] and Guidi et al. [59], who demonstrated that psychological interventions added to antidepressants significantly reduced the risk of relapse when compared with antidepressants alone. Another meta-analysis [60] that reviewed studies of time to relapse over 15 months found that the sequential delivery of a psychological intervention during and/or after tapering may be an effective relapse prevention strategy. Our study was based on full follow-up to assess the effectiveness of preventing depression relapse and used data extracted from survival curves or relapse curves for depression relapse to explore the effectiveness of different psychological interventions in preventing depression relapse at the same time points and the change in the effectiveness of psychological interventions in preventing depression relapse during the follow-up period, excluding the interference at the follow-up time.

This study had some limitations that should be taken into account when interpreting our findings. First, the number of studies for some comparisons was too low. In our network meta-analysis, some interventions (e.g., behavioral activation therapy) only included one study, and only two studies of interpersonal psychotherapy were included, leading to thinly connected networks. Second, the study evaluating the psychological intervention of behavioral activation therapy versus cognitive therapy and ADM was conducted in the setting where behavioral activation therapy was first developed, and it is possible that the investigator introduced bias into the study in favor of that modality. Therefore, considering acceptance, there were fewer withdrawals from the psychological intervention groups than from the control group, which may have caused inflated results. Third, we included all patients with depression, no matter how many episodes of depression they had suffered, which may have influenced the results for the effectiveness of MCBT in preventing depression relapse. Finally, because we used a different outcome measure than previous studies, fewer studies were included in this network meta-analysis. Therefore, we could not compare the effects of different antidepressant type, dose, and duration on patient outcomes.

In conclusion, MBCT had a continuous effect in preventing relapse of depression. CBT had the longest but not continuous effect in preventing relapse of depression. The effects of behavioral activation therapy and interpersonal therapy for the prevention of depression appeared late. All psychological interventions included in the study were more effective than supportive counseling. More evidence is needed from large comparative trials that provide long-term follow-up data.

### Supplementary information

Supplement
