# Supplementary material for: Psychological interventions for the prevention of depression relapse: systematic review and network meta-analysis
Source: Transl Psychiatry. 2023 Sep 28;13:300. doi: 10.1038/s41398-023-02604-1 (PMC10539522; doi:10.1038/s41398-023-02604-1)
Supplement: Supplementary file 1 — Supplement [file 41398_2023_2604_MOESM1_ESM.pdf]

Supplementary appendix

eAppendix1: Methods and changes to the original protocol
eTable 1: PRISMA checklist
eTable 2: Keywords in database and search result
eTable 3: Characteristics of the included studies
eTable 4: The detailed descriptions of these psychotherapies and psychological control conditions
eTable 5: League table of primary outcomes
eTable 6: Assessment of Inconsistencies
eTable 7: Sensitive analyses: League Table for limiting to studies reporting the narrowly defined diagnosis
eTable 8: Sensitive analyses: League Table for limiting to studies reporting the narrowly defined relapse
eTable 9: Sensitive analyses: League Table for limiting to studies in which the population was in remission at the time of grouping
eTable 10: Sensitive analyses: League Table for limiting to studies in which population was not the first episode of depression
eTable 11: Sensitive analyses: League Table for excluding arms that included placebo
eFigure 1: Summary and detailed risk of bias
eFigure 2: Network plot
eFigure 3: Forest plot of primary
eFigure 4: Funnel plot
eFigure 5: Transitivity assessment
eReferences
eCINeMA

eAppendix 1. Methods and changes to the original protocol

Methods

This study was conducted and reported according to the Preferred Reporting Items for Systematic Reviews and Meta-Analysis (PRISMA) guidelines for NMA.

Changes to the original protocol

1. In order to obtain more comprehensive data, we added some text words in search strategy. For example, the additional words inclue ‘Psychotherapy’, ‘Psychotherapies’, ‘Cognitive Therapy’, ‘Behavior Therapy, Cognitive’.
2. We had planned to conducted an NMA of trials comparing cognitive behavioural, interpersonal, psychodynamic, problem-solving, behavioural activation, life-review and third wave therapies and non-directive supportive counselling with each other and with treat-as-usual, antidepressant and pill placebo control conditions. However, we found there were not enough available data of psychodynamic, problem-solving, life-review and metacognitive therapy for network analysis after data extraction. Thus, we only analysed cognitive behavioural therapy (CBT), mindfulness based cognitive behavioural therapy (MBCT), interpersonal therapy, behavioural active therapy or supportive counselling.
3. We also added sensitivity analyses (i) and (ii), (iv) which were not in the original protocol to do justice to the heterogeneity of the identified studies and to more finely assess the robustness of our primary analyses.

eTable 1. PRISMA checklist

PRISMA NMA Checklist of Items to Include When Reporting A Systematic Review Involving a Network Meta-analysis

Section/Topic	Item #	Checklist Item	Reported on Page #
**TITLE**			
**Title**	1	Identify the report as a systematic review incorporating a network meta-analysis (for related form of meta-analysis).	1
**ABSTRACT**			
**Structured summary**	2	Provide a structured summary including, as applicable:<br>**Background:** main objective<br>**Methods:** data sources; study eligibility criteria, participants, and interventions; study appraisal; and *synthesis methods, such as network meta-analysis*.<br>**Results:** number of studies and participants identified; summary estimates with corresponding confidence/credible intervals; *treatment rankings may also be discussed. Authors may choose to summarize pairwise comparisons against a chosen treatment included in their analyses for brevity.*<br>**Discussion/Conclusions:** limitations; conclusions and implications of findings.<br>**Other:** primary source of funding; systematic review registration number with registry name.	2
**INTRODUCTION**			
**Rationale**	3	Describe the rationale for the review in the context of what is already known*, including mention of why a network meta-analysis has been conducted*.	3
**Objectives**	4	Provide an explicit statement of questions being addressed, with reference to participants, interventions, comparisons, outcomes, and study design (PICOS).	3
**METHODS**			
**Protocol and registration**	5	Indicate whether a review protocol exists and if and where it can be accessed (e.g., Web address); and, if available, provide registration information, including registration number.	5
**Eligibility criteria**	6	Specify study characteristics (e.g., PICOS, length of follow-up) and report characteristics (e.g., years considered, language, publication status) used as criteria for eligibility, giving rationale. *Clearly describe eligible treatments included in the treatment network, and note whether any have been clustered or merged into the same node (with justification).*	4
**Information sources**	7	Describe all information sources (e.g., databases with dates of coverage, contact with study authors to identify additional studies) in the search and date last searched.	4
**Search**	8	Present full electronic search strategy for at least one database, including any limits used, such that it could be repeated.	Supplement
**Study selection**	9	State the process for selecting studies (i.e., screening, eligibility, included in systematic review, and, if applicable, included in the meta-analysis).	4, Figure 1 and Supplement
**Data collection process**	10	Describe method of data extraction from reports (e.g., piloted forms, independently, in duplicate) and any processes for obtaining and confirming data from investigators.	4 and Supplement
**Data items**	11	List and define all variables for which data were sought (e.g., PICOS, funding sources) and any assumptions and simplifications made.	4 and Supplement

**Geometry of the network**	S1	Describe methods used to explore the geometry of the treatment network under study and potential biases related to it. This should include how the evidence base has been graphically summarized for presentation, and what characteristics were compiled and used to describe the evidence base to readers.	4-5
**Risk of bias within individual studies**	12	Describe methods used for assessing risk of bias of individual studies (including specification of whether this was done at the study or outcome level), and how this information is to be used in any data synthesis.	5
**Summary measures**	13	State the principal summary measures (e.g., risk ratio, difference in means). *Also describe the use of additional summary measures assessed, such as treatment rankings and surface under the cumulative ranking curve (SUCRA) values, as well as modified approaches used to present summary findings from meta-analyses.*	5
**Planned methods of analysis**	14	Describe the methods of handling data and combining results of studies for each network metaanalysis. This should include, but not be limited to:<br>• *Handling of multi-arm trials;*<br>• *Selection of variance structure;*<br>• *Selection of prior distributions in Bayesian analyses; and*<br>• *Assessment of model fit.*	4-5
**Assessment of Inconsistency**	S2	Describe the statistical methods used to evaluate the agreement of direct and indirect evidence in the treatment network(s) studied. Describe efforts taken to address its presence when found.	4-5
**Risk of bias across studies**	15	Specify any assessment of risk of bias that may affect the cumulative evidence (e.g., publication bias, selective reporting within studies).	5 and Supplement
**Additional analysis**	16	Describe methods of additional analyses if done, indicating which were pre-specified. This may include, but not be limited to, the following:<br>• *Sensitivity or subgroup analyses;*<br>• *Meta-regression analyses;*<br>• *Alternative formulations of the treatment network; and*<br>• *Use of alternative prior distributions for Bayesian analyses (if applicable)*	4-5 and Supplement
**RESULTS**			
**Study selection**	17	Give numbers of studies screened, assessed for eligibility, and included in the review, with reasons for exclusions at each stage, ideally with a flow diagram.	5
**Presentation of network structure**	S3	Provide a network graph of the included studies to enable visualization of the geometry of the treatment network.	Figure 2 and Supplement
**Summary of network geometry**	S4	Provide a brief overview of characteristics of the treatment network. This may include commentary on the abundance of trials and randomized patients for the different interventions and pairwise comparisons in the network, gaps of evidence in the treatment network, and potential biases reflected by the network structure.	5-6 and Supplement
**Study characteristics**	18	For each study, present characteristics for which data were extracted (e.g., study size, PICOS, follow-up period) and provide the citations.	5-6 and Supplement
**Risk of bias within studies**	19	Present data on risk of bias of each study and, if available, any outcome level assessment.	5-6 and Supplement
**Results of individual studies**	20	For all outcomes considered (benefits or harms), present, for each study: 1) simple summary data for each intervention group, and 2) effect estimates and confidence intervals. *Modified approaches may be needed to deal with information from larger networks.*	6-7, Figure 3-6 and Supplement

**Synthesis of results**	21	Present results of each meta-analysis done, including confidence/credible intervals. In larger networks, authors may focus on comparisons versus a particular comparator *(e.g., placebo or standard care),* with full findings presented in an appendix. *League tables and forest plots may be considered to summarize pairwise comparisons.* If additional summary measures were explored (such as treatment rankings), these should also be presented.	Figure 3-6 and Supplement
**Exploration for inconsistency**	S5	Describe results from investigations of inconsistency. This may include such information as measures of model fit to compare consistency and inconsistency models, P values from statistical tests, or summary of inconsistency estimates from different parts of the treatment network.	6-8 and Supplement
**Risk of bias across studies**	22	Present results of any assessment of risk of bias across studies for the evidence base being studied.	6 and Supplement
**Results of additional analysis**	23	Give results of additional analyses, if done (e.g., sensitivity or subgroup analyses, meta-regression analyses, *alternative network geometries studied, alternative choice of prior distributions for Bayesian analyses,* and so forth).	7-8 and Supplement
**DISCUSSION**			
**Summary of evidence**	24	Summarize the main findings, including the strength of evidence for each main outcome; consider their relevance to key groups (e.g., healthcare providers, users, and policy-makers).	8
**Limitations**	25	Discuss limitations at study and outcome level (e.g., risk of bias), and at review level (e.g., incomplete retrieval of identified research, reporting bias). *Comment on the validity of the assumptions, such as transitivity and consistency. Comment on any concerns regarding network geometry (e.g., avoidance of certain comparisons).*	9-10
**Conclusions**	26	Provide a general interpretation of the results in the context of other evidence, and implications for future research.	10
**FUNDING**			
**Funding**	27	Describe sources of funding for the systematic review and other support (e.g., supply of data); role of funders for the systematic review. This should also include information regarding whether funding has been received from manufacturers of treatments in the network and/or whether some of the authors are content experts with professional conflicts of interest that could affect use of treatments in the network.	11

PICOS = population, intervention, comparators, outcomes, study design.
* Text in italics indicates wording specific to reporting of network meta-analyses that has been added to guidance from the PRISMA statement.

eTable 2: Keywords in database and search result

We plan to search PubMed, Embase, PsycINFO via Ovid, and Cochrane Library from inception using the following terms:

(Depressive Disorder or Depressive Disorders or Disorder, Depressive or Disorders, Depressive or Neurosis, Depressive or Depressive Neuroses or Depressive Neurosis or Neuroses, Depressive or Depressions, Endogenous or Depression, Endogenous or Endogenous Depression or Endogenous Depressions or Depressive Syndrome or Depressive Syndromes or Syndrome, Depressive or Syndromes, Depressive or Depression, Neurotic or Depressions, Neurotic or Neurotic Depression or Neurotic Depressions or Melancholia or Melancholias or Unipolar Depression or Depression, Unipolar or Depressions, Unipolar or Unipolar Depressions) in Abstract AND (Psychotherapy or Psychotherapies or Psychotherapists or Psychotherapist or Clinical Psychotherapists or Clinical Psychotherapist or Psychotherapist, Clinical or Psychotherapists, Clinical or Cognitive Behavioral Therapy or Behavioral Therapies, Cognitive or Behavioral Therapy, Cognitive or Cognitive Behavioral Therapies or Therapies, Cognitive Behavioral or Therapy, Cognitive Behavioral or Behavior Therapy, Cognitive or Cognitive Behavior Therapy or Cognitive Behaviour Therapy or Behaviour Therapies, Cognitive or Behaviour Therapy, Cognitive or Cognitive Behaviour Therapies or Therapies, Cognitive Behaviour or Therapy, Cognitive Behaviour or Cognitive Therapy or Therapy, Cognitive Behavior or Behavior Therapies, Cognitive or Cognitive Behavior Therapies or Therapies, Cognitive Behavior or Cognitive Psychotherapy or Cognitive Psychotherapies or Psychotherapies, Cognitive or Psychotherapy, Cognitive or Therapy, Cognitive or Cognitive Therapies or Therapies, Cognitive or Therapy, Cognition or Cognition Therapy or Cognition Therapies or Therapies, Cognition or interpersonal psychotherapy or Mindfulness-based cognitive therapy or problem-solving therapy or non-directive supportive counseling or life-review therapy or psychodynamic therapy) in Abstract

Numbers of citations by each database

Databases, trial registers and other sources	Citations
**Databases:**	
PubMed	3514
Cochrane Library	6608
Embase	3179
PsycINFO	3472
**Other sources:**	8
**Total citations:**	16736

eReferences

1. Bockting, C.L., et al., *Preventing relapse/recurrence in recurrent depression with cognitive therapy: a randomized controlled trial.* J Consult Clin Psychol, 2005. 73(4): p. 647-57.
2. Bockting, C.L.H., et al., *Effectiveness of preventive cognitive therapy while tapering antidepressants versus maintenance antidepressant treatment versus their combination in prevention of depressive relapse or recurrence (DRD study): a three-group, multicentre, randomised controlled trial.* Lancet Psychiatry, 2018. 5(5): p. 401-410.
3. Dobson, K.S., et al., *Randomized trial of behavioral activation, cognitive therapy, and antidepressant medication in the prevention of relapse and recurrence in major depression.* Journal of consulting and clinical psychology, 2008. 76(3): p. 468 - 477.
4. Fava, G.A., et al., *Six-year outcome for cognitive behavioral treatment of residual symptoms in major depression.* Am J Psychiatry, 1998. 155(10): p. 1443-5.
5. Godfrin, K.A. and C. van Heeringen, *The effects of mindfulness-based cognitive therapy on recurrence of depressive episodes, mental health and quality of life: A randomized controlled study.* Behaviour Research and Therapy, 2010. 48(8): p. 738-746.
6. Holländare, F., et al., *Two-year outcome of internet-based relapse prevention for partially remitted depression.* Behav Res Ther, 2013. 51(11): p. 719-22.
7. Hollon, S.D., et al., *Prevention of relapse following cognitive therapy vs medications in moderate to severe depression.* Arch Gen Psychiatry, 2005. 62(4): p. 417-22.
8. Huijbers, M.J., et al., *Adding mindfulness-based cognitive therapy to maintenance antidepressant medication for prevention of relapse/recurrence in major depressive disorder: Randomised controlled trial.* J Affect Disord, 2015. 187: p. 54-61.
9. Jarrett, R.B., et al., *Preventing recurrent depression using cognitive therapy with and without a continuation phase: a randomized clinical trial.* Arch Gen Psychiatry, 2001. 58(4): p. 381-8.
10. Jarrett, R.B., et al., *Preventing depressive relapse and recurrence in higher-risk cognitive therapy responders: a randomized trial of continuation phase cognitive therapy, fluoxetine, or matched pill placebo.* JAMA Psychiatry, 2013. 70(11): p. 1152-60.
11. Kennedy, J.C., et al., *Follow-up of monotherapy remitters in the PReDICT study: Maintenance treatment outcomes and clinical predictors of recurrence.* Journal of Consulting and Clinical Psychology, 2018. 86(2): p. 189-199.

12. Kuyken, W., et al., *Mindfulness-based cognitive therapy to prevent relapse in recurrent depression.* Journal of consulting and clinical psychology, 2008. 76(6): p. 966 - 978.
13. Kuyken, W., et al., *Effectiveness and cost-effectiveness of mindfulness-based cognitive therapy compared with maintenance antidepressant treatment in the prevention of depressive relapse or recurrence (PREVENT): a randomised controlled trial.* Lancet (london, england), 2015. 386(9988): p. 63 - 73.
14. Lemmens, L.H.J.M., et al., *Long-term outcomes of acute treatment with cognitive therapy v. interpersonal psychotherapy for adult depression: follow-up of a randomized controlled trial.* Psychological medicine, 2019. 49(3): p. 465-473.
15. Morokuma, I., et al., *Psychoeducation for major depressive disorders: a randomised controlled trial.* Psychiatry research, 2013. 210(1): p. 134 - 139.
16. Paykel, E.S., et al., *Duration of relapse prevention after cognitive therapy in residual depression: follow-up of controlled trial.* Psychol Med, 2005. 35(1): p. 59-68.
17. Perlis, R.H., et al., *Effects of adding cognitive therapy to fluoxetine dose increase on risk of relapse and residual depressive symptoms in continuation treatment of major depressive disorder.* J Clin Psychopharmacol, 2002. 22(5): p. 474-80.
18. Peselow, E.D., et al., *Prophylactic efficacy of fluoxetine, escitalopram, sertraline, paroxetine, and concomitant psychotherapy in major depressive disorder: outcome after long-term follow-up.* Psychiatry Res, 2015. 225(3): p. 680-6.
19. Segal, Z.V., et al., *Antidepressant monotherapy vs sequential pharmacotherapy and mindfulness-based cognitive therapy, or placebo, for relapse prophylaxis in recurrent depression.* Arch Gen Psychiatry, 2010. 67(12): p. 1256-64.
20. Shallcross, A.J., et al., *Relapse/Recurrence Prevention in Major Depressive Disorder: 26-Month Follow-Up of Mindfulness-Based Cognitive Therapy Versus an Active Control.* Behavior therapy, 2018. 49(5): p. 836 - 849.
21. Shea, M.T., et al., *Course of depressive symptoms over follow-up. Findings from the National Institute of Mental Health Treatment of Depression Collaborative Research Program.* Archives of general psychiatry, 1992. 49(10): p. 782 - 787.
22. Simons, A.D., et al., *Cognitive therapy and pharmacotherapy for depression. Sustained improvement over one year.* Arch Gen Psychiatry, 1986. 43(1): p. 43-8.

23. Stangier, U., et al., *Maintenance cognitive-behavioral therapy and manualized psychoeducation in the treatment of recurrent depression: a multicenter prospective randomized controlled trial.* Am J Psychiatry, 2013. 170(6): p. 624-32.
24. Klein, N.S., et al., *No Sustainable Effects of an Internet-Based Relapse Prevention Program over 24 Months in Recurrent Depression: Primary Outcomes of a Randomized Controlled Trial.* Psychother Psychosom, 2018. 87(1): p. 55-57.
25. Ma, S.H. and J.D. Teasdale, *Mindfulness-based cognitive therapy for depression: replication and exploration of differential relapse prevention effects.* J Consult Clin Psychol, 2004. 72(1): p. 31-40.

eTable 3: Characteristics of the included studies

	Interventions* (total number of patients)	Women (%)	Mean age	Follow-up Duration (months)	Diagnosis (criteria)	Relapse (criteria)	Number of episodes
Bockting 2005	CBT (n=88), TAU (n=84)	73	45	24m	DSM–IV MDD	DSM–IV MDD	⩾2
Bocting 2018	CBT+ADM (n=104), ADM (n=100)	65	47	24m	DSM–IV MDD	DSM–IV MDD	⩾2
Dobson 2008	CBT (n=30), BA (n=27), ADM (n=28), PLA (n=21)	69	40	24m	DSM–IV MDD	Either HRSD scores of 14 or greater, or PSRs of 5 or greater, for two successive weeks.	⩾1
Fava 1998	ADM+CBT (n=20), ADM (n=20)	68	46	72m	The patients' diagnoses were established by the consensus of a psychiatrist (G.A.F.) and a clinical psychologist (S.G.) independently using the Schedule for Affective Disorders and Schizophrenia.	An RDC-defined episode of major depression.	⩾1
Godfrin 2010	MBCT (n=52), TAU (n=54)	81	46	14m	DSM–IV MDD	DSM–IV MDD	⩾3
Hollandare 2013	CBT (n=42), SUP (n=42)	85	45	24m	DSM–IV MDD	DSM–IV MDD	⩾1

Hollon 2005	CBT (n=35), ADM (n=34), PLA （n=35）	59	40	12m	DSM–IV MDD	⩾ 14 on the HDRS for 2 consecutive weeks.	⩾1
Huijbers 2015	MBCT+ADM (n=33), ADM (n=35)	72	52	15m	DSM–IV MDD	with the SCID-I by trained research assistants	⩾3
Jarrett 2001	CBT (n=41), SUP (n=43)	61	43	16m	DSM–IV MDD	DSM–IV MDD	⩾1
Jarrett 2013	CBT (n=86), ADM (n=86), PLA (n=69)	67	43	24m	DSM–IV MDD	DSM–IV MDD	⩾2
Kennedy 2018	CBT (n=23), ADM (n=71)	50	38	21m	DSM–IV MDD	A patient meeting any one of the four following criteria: 1) meeting criteria for a major depressive episode based on a LIFE score of 3 or greater; 2) a 17-item HAM-D ≥ 14 for two consecutive weeks (patients with an HAM-D ≥ 14 at a follow-up visit were asked to return the following week for an additional rating); 3) a 17-item HAM-D ≥ 14 at any follow-up visit and at which time the patient requested an immediate change in treatment; and 4) high risk of suicide, as determined by the study psychiatrist .	⩾1
Klein 2018	CBT (n=132), TAU (n=132)	75	46	24m	DSM–IV MDD	DSM–IV MDD	⩾2

Kuyken 2008	MBCT (n=61), ADM (n=62)	76	49	15m	DSM–IV MDD	DSM–IV MDD	⩾3
Kuyken 2015	MBCT (n=212), ADM (n=212)	77	50	24m	DSM–IV MDD	DSM–IV MDD	⩾3
Lemmens 2019	CBT (n=69), IPT (n=65)	66	41	17m	DSM–IV MDD	Relapse on the BDI-II was defined as losing >=50% of the improvement that occurred over the course of treatment at any point during the follow-up.	⩾1
Ma 2004	MBCT (n=28), TAU (n=27)	76	45	12m	DSM–IV MDD	DSM–IV MDD	⩾2 (survival curve>=3
Morokuma 2013	SUP+ADM (n=19), ADM (n=15)	56	43	9m	DSM–IV MDD	DSM–IV MDD	⩾1
Paykel 2005	CBT+ADM (n=70), ADM (n=65)	50	43	72m	DSM–III MDD	DSM–III MDD	⩾1
Perlis 2002	CBT+ADM (n=66), ADM (n=66)	55	40	7m	DSM–III MDD	DSM–III MDD	⩾3
Peselow 2015	CBT+ADM (n=88), ADM (n=299)	59	32	144m	DSM–IV MDD	DSM–IV MDD	⩾1

Segal 2010	MBCT (n=26), ADM (n=28), PLA (n=30)	63	44	18m	DSM–IV MDD	DSM–IV MDD	⩾3
Shallcross 2018	MBCT (n=46), SUP (n=46)	76	35	26m	DSM–IV MDD	DSM–IV MDD	⩾1
Shea 1992	CBT (n=59), IPT (n=61), ADM (n=57), PLA (n=62)	70	35	18m	RDC MDD<br>HRSD>=14	The conventional LIFE-II definition of relapse was used: two consecutive weeks of symptoms meeting RDC for definite MDD (5 or 6 on the PSR scale).	⩾1
Simons 1986	CBT (n=19), CBT+ADM (n=18), ADM (n=16)	52	34	12m	DSM–III MDD	BDI scores of 16 or higher	⩾1
Stangier 2013	CBT+ADM (n=90), SUP+ADM (n=90)	72	49	12m	DSM–IV MDD	DSM–IV MDD	⩾3

* names of psychological interventions have been standardized, according to the intervention description.

eTable 4: The detailed descriptions of these psychotherapies and psychological control conditions

Interventions	Abbreviation	Description
Psychotherapeutic Intervention:		
Behavioural activation therapy	BA	We considered an intervention to be behavioral activation when the registration of pleasant activities and the increase of positive interactions between a person and his or her environment were the core elements of the treatment. Social skills training could be a part of the intervention.
Cognitive behavior therapy	CBT	In CBT the therapists focus on the impact a patient's present dysfunctional thoughts have on current behavior and future functioning. CBT is aimed at evaluating, challenging and modifying a patient's dysfunctional beliefs (cognitive restructuring). In this form of treatment the therapist mostly emphasizes homework assignments and outside-of-session activities. Therapists exert an active influence over therapeutic interactions and topics of discussion, use a psycho educational approach, and teach patients new ways of coping with stressful situations.
Interpersonal therapy	IPT	IPT is a brief and highly structured manual-based psychotherapy that addresses interpersonal issues in depression, to the exclusion of all other foci of clinical attention.
Mindfulness-based cognitive therapy	MBCT	Mindfulness-Based Cognitive Therapy (MBCT) is using guided meditations and exercises from CBT, participants were taught skills to identify cognitive distortions and to disengage from depression-focused ruminative thinking patterns through the use of non-judgmental and present-focused awareness.

Life review therapy	LRT	Reminiscence is a naturally occurring process of recalling the past, that is hypothesized to resolve conflicts from the past and make up the balance of one's life. Since the beginning of the 1970s, reminiscence has been used by therapists as a specific treatment of depression in older adults. In these life review therapies the patients work through the memories of all phases in their life with the aim of re-evaluation of their life, resolving conflicts or assessing adaptive coping-responses. We defined life review therapies as all therapies that are aimed at the systematic evaluation of the lives of participants.
Psychodynamic therapy	DYN	The primary objective in (short-term) psychodynamic therapy is to enhance the patient's understanding, awareness and insight about repetitive conflicts (intra psychic and intrapersonal). An assumption in psychodynamic therapy is that a patient's childhood experiences, past unresolved conflicts, and historical relationships significantly affect a person's present life situation. In this form of treatment the therapist concentrates on the patient's past, unresolved conflicts, historical relationships and the impact these have on a patient's present functioning. Furthermore, in psychodynamic therapy the therapists explore a patient's wishes, dreams, and fantasies. The time limitations and the focal explorations of the patient's life and emotions distinguish psychodynamic therapy from psychoanalytic psychotherapy. We did not identify subtypes of psychodynamic therapy.
Problem-solving therapy	PST	We defined PST as a psychological intervention in which the following elements had to be included: definition of personal problems, generation of multiple solutions to each problem, selection of the best solution, the working out of a systematic plan for this solution, and evaluation as to whether the solution has resolved the problem.

Supportive counselling	SUP	We defined Supportive counselling as any unstructured therapy without specific psychological techniques other than those common to all approaches such as helping people to ventilate their experiences and emotions and offering empathy. It is not aimed at solutions, or acquiring new skills. It assumes that relief from personal problems may be achieved through discussion with others.
Control conditions:		
Placebo	PLA	PLA is a control condition that was regarded as inactive by the researchers, but was to be the participants.
Treatment as usual	TAU	TAU is not considered to be structured psychotherapy, but may have some treatment effects.
Antidepressant	ADM	ADM is a control condition in which the participants receive any kind of antidepressant.

eFigure 1A: Risk of Bias Summary

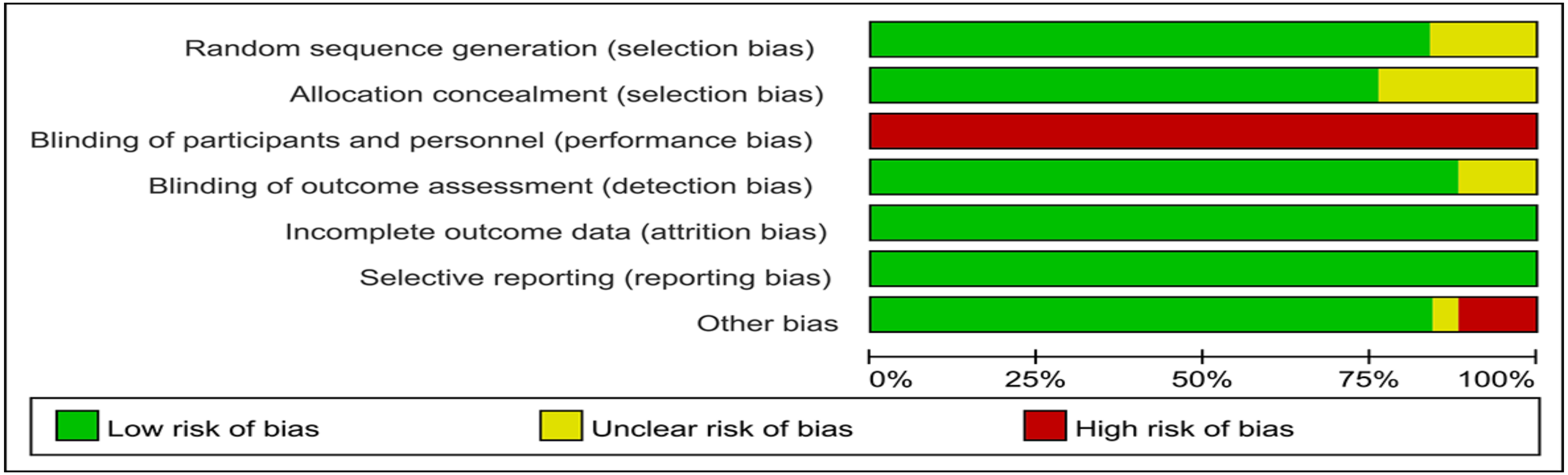

eFigure 1B: Risk of Bias Graph

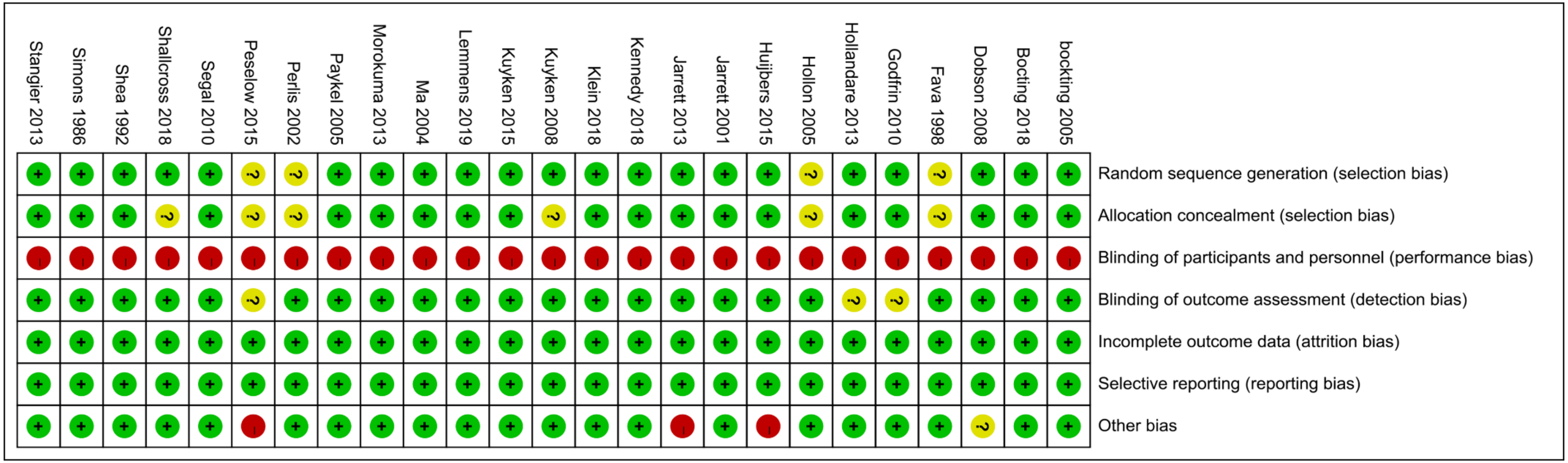

Note

Legend for network plots: lines link treatments with direct comparisons in trials; thickness of lines corresponds to the number of trials evaluating the comparison; size of the nodes corresponds to the number of trials investigating the treatment.

Legend for forest-plots: effect sizes are from the network-meta-analysis. The type of effect size measure (OR=odds ratio) can be seen on top right.
Order of treatments is according to the surface under the cumulative ranking. Reference is ADM.
The direction of the effect is indicated below the y-axis. In general, effect estimates to the left are in favor of psychological treatment.

Legend for league tables: Odds risks (ORs) and 95% confidence intervals (CIs) are reported. Results of the network meta-analysis are reported in the lower left part of the table. ORs higher than 1 favour the column-defining treatment. Statistically significant results are highlighted in grey.

Abbreviations: ADM=antidepressant. BA=behavioural active therapy. CBT=cognitive behavioral therapy. CBT+ADM=cognitive behavioral therapy combined with antidepressant. IPT=interpersonal therapy. MBCT=mindfulness-based cognitive therapy. MBCT+ADM=mindfulness-based cognitive therapy combined with antidepressant. PLA=placebo. SUP=supportive counselling. SUP+ADM=supportive counselling combined with antidepressant. TAU=treatment as usual.

eFigure 2A: Network Plot (3-month follow-up timepoint)

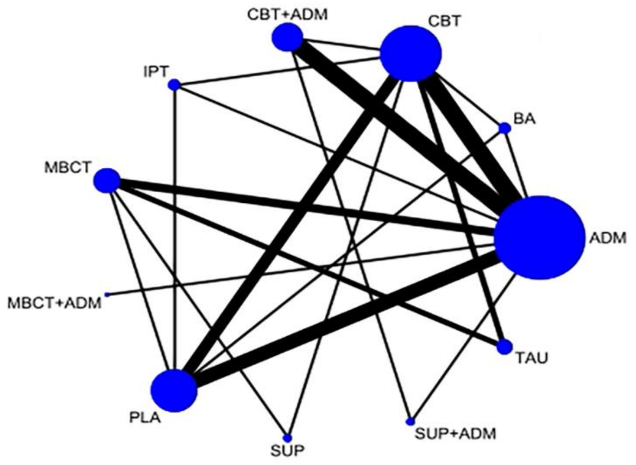

eFigure 2B: Network Plot (6-month follow-up timepoint)

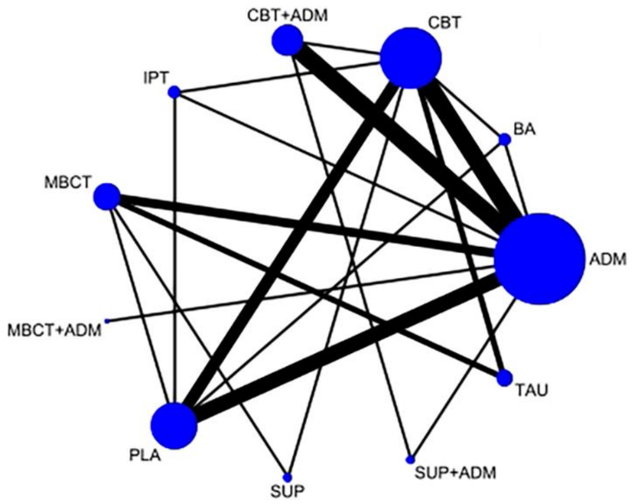

eFigure 2C: Network Plot (9-month follow-up timepoint)

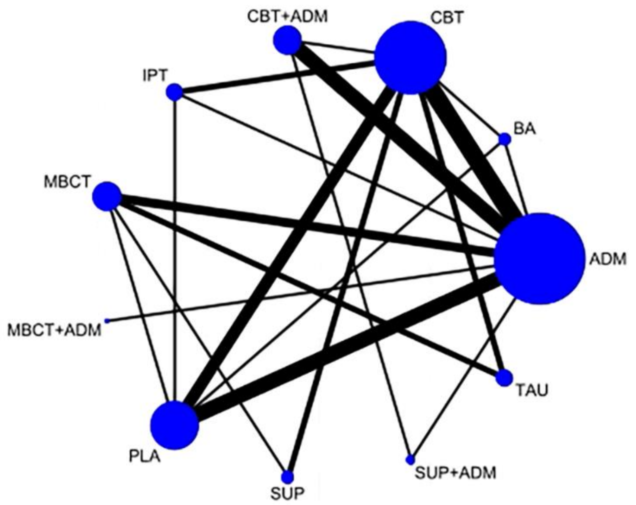

eFigure 2D: Network Plot (12-month follow-up timepoint)

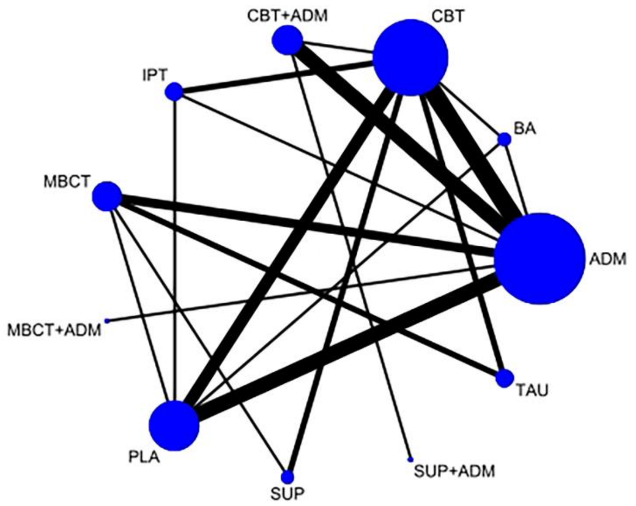

eFigure 2E: Network Plot (15-month follow-up timepoint)

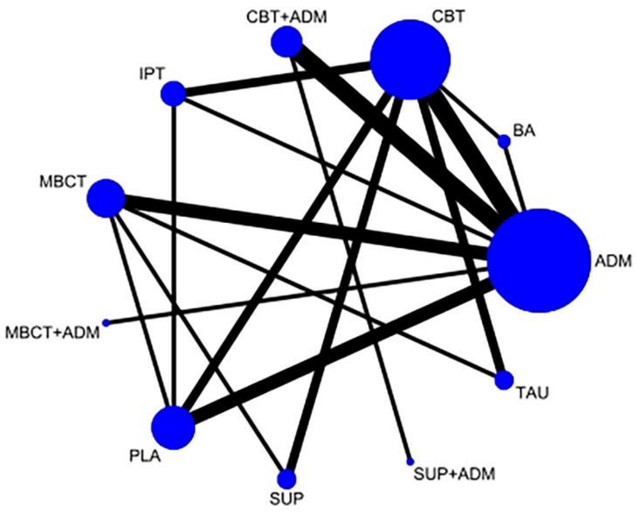

eFigure 2F: Network Plot (18-month follow-up timepoint)

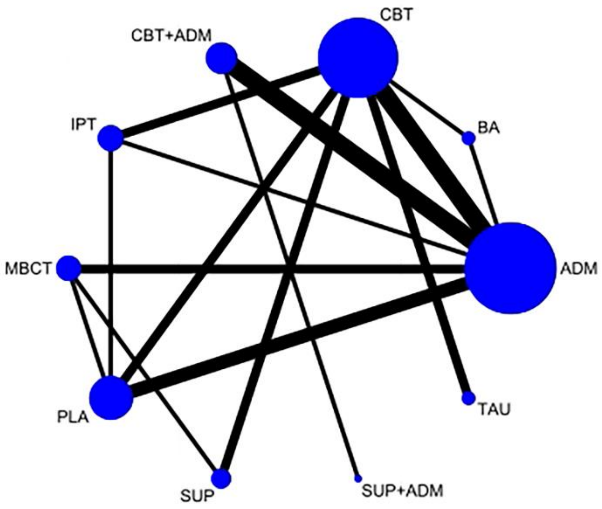

eFigure 2G: Network Plot (21-month follow-up timepoint)

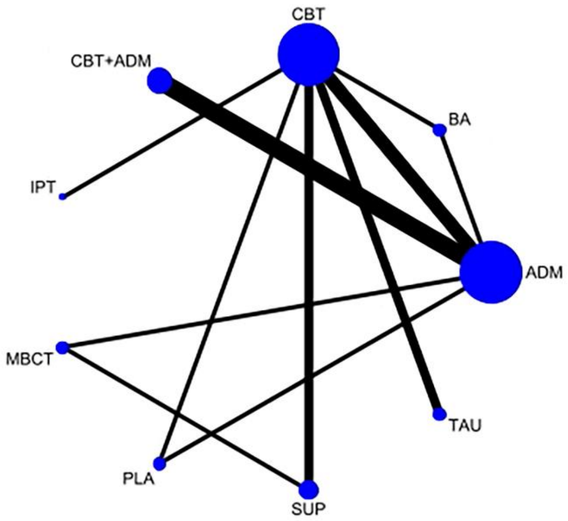

eFigure 2H: Network Plot (24-month follow-up timepoint)

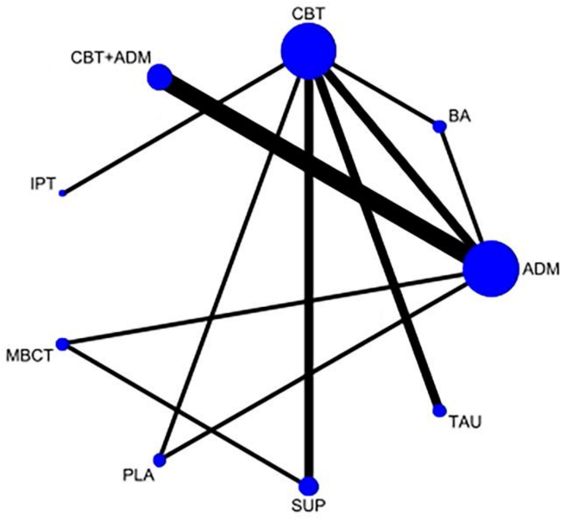

eFigure 3A: Forest plot of primary (3-month follow-up timepoint)

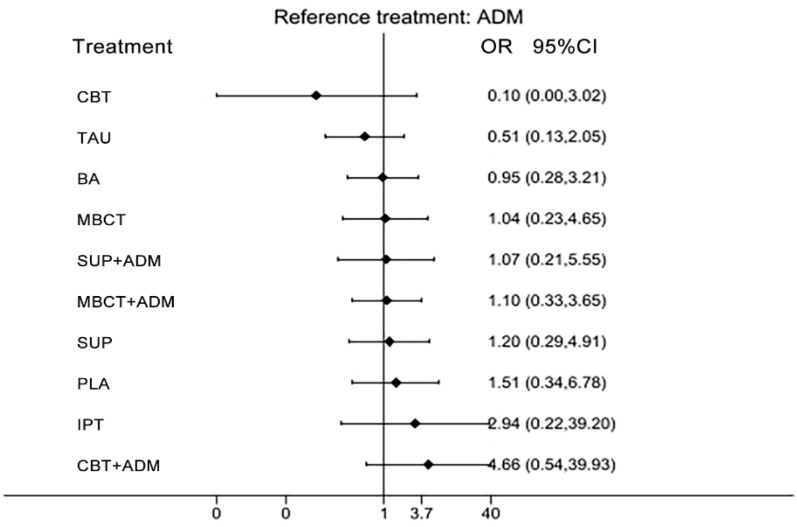

eFigure 3B: Forest plot of primary (6-month follow-up timepoint)

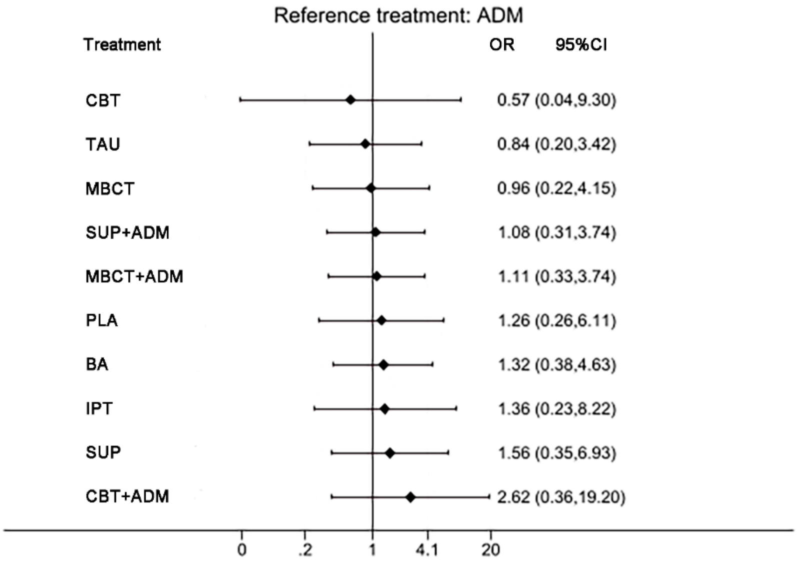

eFigure 3C: Forest plot of primary (9-month follow-up timepoint)

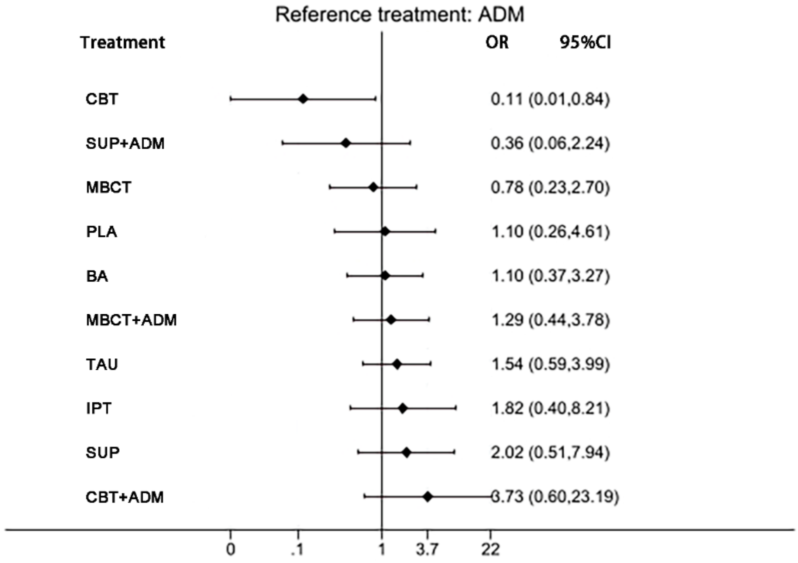

eFigure 3D: Forest plot of primary (12-month follow-up timepoint)

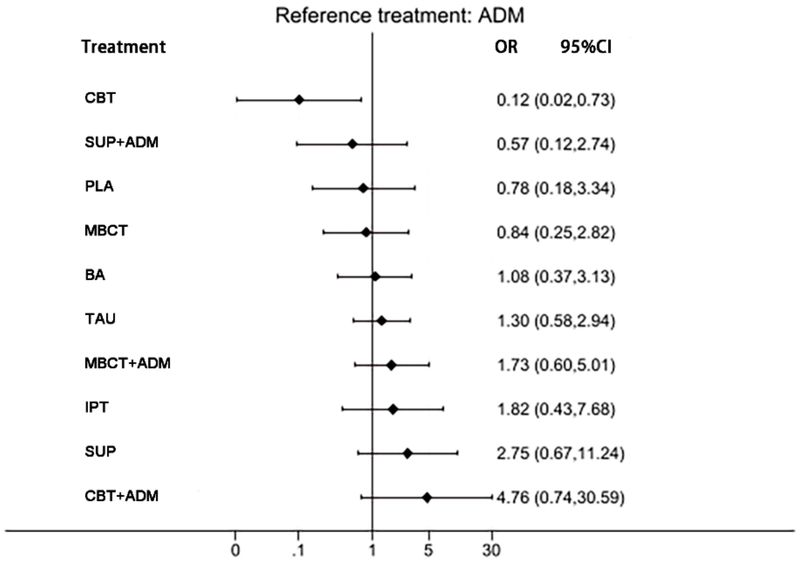

eFigure 3E: Forest plot of primary (15-month follow-up timepoint)

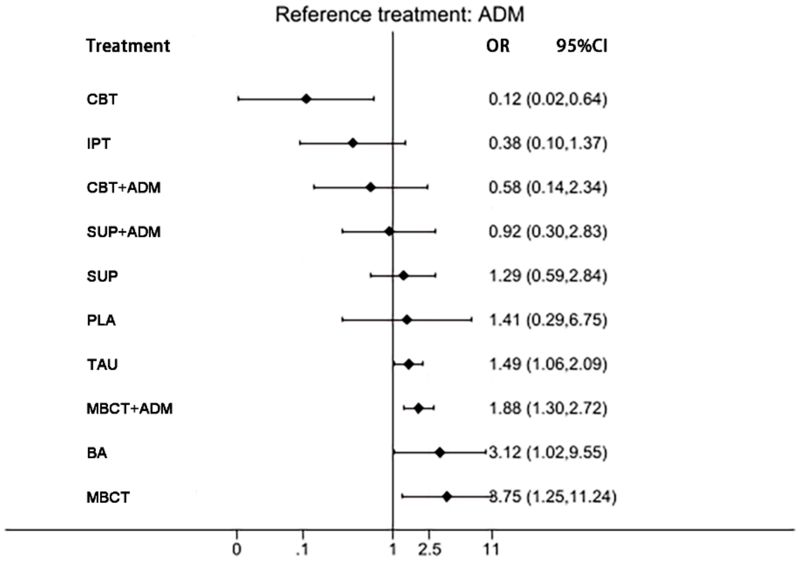

eFigure 3F: Forest plot of primary (18-month follow-up timepoint)

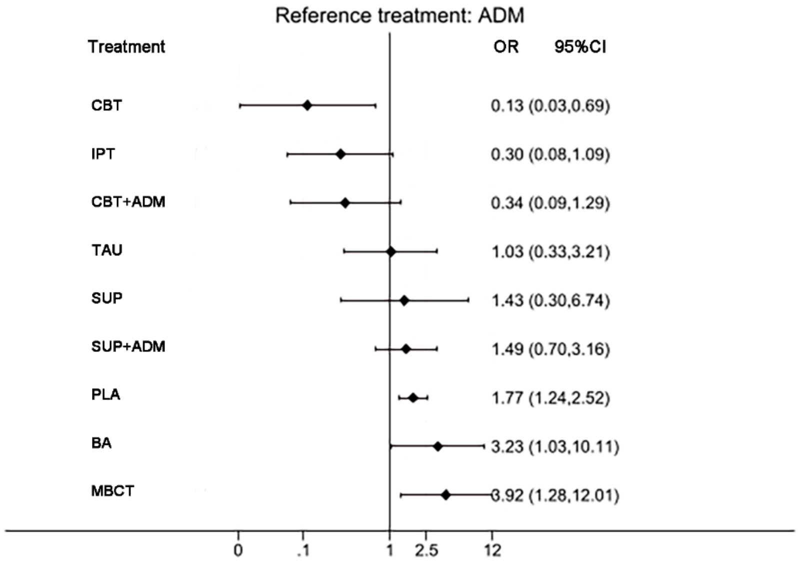

eFigure 3G: Forest plot of primary (21-month follow-up timepoint

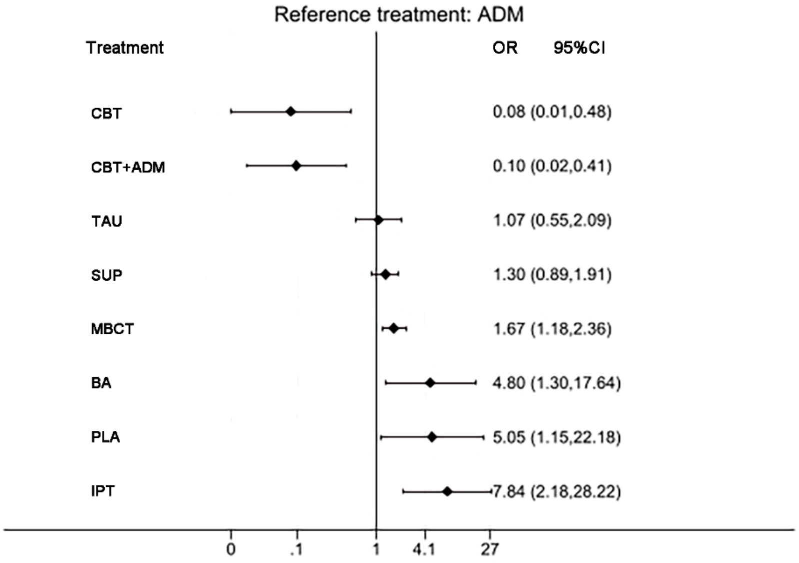

eFigure 3H: Forest plot of primary (24-month follow-up timepoint)

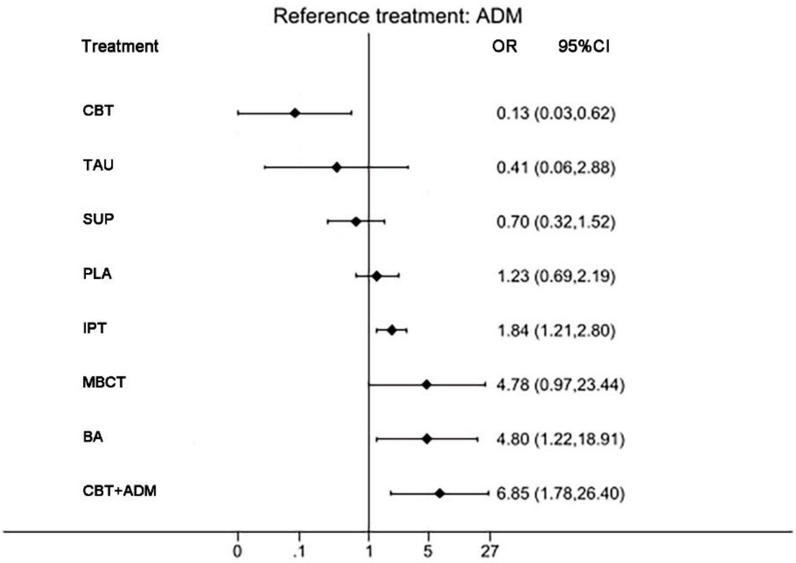

eTable 5A: League Table for 3-month follow-up timepoint

SUP+ADM										
2.86 (0.07,119.46)	MBCT+ADM									
8.61 (0.41,183.03)	3.01 (0.31,29.00)	MBCT								
13.93 (0.54,356.71)	4.87 (0.40,59.98)	1.62 (0.44,6.00)	CBT							
12.72 (0.46,353.35)	4.44 (0.32,60.79)	1.48 (0.33,6.63)	0.91 (0.14,5.82)	CBT+ADM						
14.21 (0.46,438.77)	4.97 (0.32,77.58)	1.65 (0.29,9.25)	1.02 (0.13,7.81)	1.12 (0.13,9.73)	IPT					
16.02 (0.62,411.82)	5.60 (0.45,69.32)	1.86 (0.50,6.97)	1.15 (0.34,3.84)	1.26 (0.20,8.09)	1.13 (0.15,8.69)	BA				
1.07 (0.21,5.55)	1.10 (0.33,3.65)	1.04 (0.23,4.65)	0.10 (0.00,3.02)	4.66 (0.54,39.93)	2.94 (0.22,39.20)	0.95 (0.28,3.21)	ADM			
31.55 (1.18,845.07)	11.02 (0.84,143.94)	3.66 (0.89,15.15)	2.26 (1.31,3.90)	2.48 (0.36,17.10)	2.22 (0.27,18.27)	1.97 (0.52,7.40)	1.95 (0.49,7.79)	TAU		
34.34 (1.33,883.31)	12.00 (0.97,148.72)	3.99 (1.06,14.97)	2.46 (0.74,8.26)	2.70 (0.42,17.36)	2.42 (0.31,18.65)	2.14 (0.63,7.26)	0.66 (0.15,2.97)	1.09 (0.29,4.10)	PLA	
75.99 (2.04,2825.71)	26.56 (1.35,521.50)	8.82 (1.11,69.83)	5.45 (1.10,27.01)	5.98 (0.52,69.09)	5.35 (0.40,71.25)	4.74 (0.64,35.19)	0.83 (0.20,3.41)	2.41 (0.44,13.05)	2.21 (0.30,16.45)	SUP

eTable 5B: League Table for 6-month follow-up timepoint

SUP+ADM											
8.73 (0.80,95.45)	MBCT										
10.13 (0.64,159.87)	1.16 (0.23,5.77)	CBT+ADM									
11.25 (0.76,166.76)	1.29 (0.29,5.74)	1.11 (0.15,8.46)	MBCT+ADM								
11.94 (0.85,166.90)	1.37 (0.34,5.46)	1.18 (0.17,8.30)	1.06 (0.16,6.83)	BA							
14.17 (1.03,194.21)	1.62 (0.42,6.24)	1.40 (0.20,9.59)	1.26 (0.20,7.88)	1.19 (0.34,4.13)	CBT						
14.58 (1.05,202.58)	1.67 (0.42,6.59)	1.44 (0.21,10.05)	1.30 (0.20,8.26)	1.22 (0.21,7.15)	1.03 (0.18,5.84)	IPT					
1.08 (0.31,3.74)	0.96 (0.22,4.15)	2.62 (0.36,19.20)	1.11 (0.33,3.74)	1.32 (0.38,4.63)	0.57 (0.04,9.30)	1.36 (0.23,8.22)	ADM				
24.36 (1.66,356.57)	2.79 (0.64,12.15)	2.41 (0.32,18.02)	2.16 (0.32,14.86)	2.04 (0.51,8.12)	1.72 (0.95,3.11)	1.67 (0.27,10.46)	1.20 (0.29,4.90)	TAU			
37.80 (2.67,535.00)	4.33 (1.06,17.71)	3.73 (0.52,26.72)	3.36 (0.51,21.99)	3.17 (0.85,11.78)	2.67 (0.75,9.52)	2.59 (0.44,15.44)	0.80 (0.16,3.86)	1.55 (0.38,6.32)	PLA		
113.38 (5.10,2518.06)	12.99 (1.53,110.28)	11.20 (0.88,142.36)	10.08 (0.85,119.63)	9.50 (1.19,75.87)	8.00 (1.52,42.15)	7.77 (0.70,85.97)	0.64 (0.14,2.86)	4.66 (0.80,27.18)	3.00 (0.37,24.33)	SUP	

eTable 5C: League Table for 9-month follow-up timepoint

SUP+ADM										
8.74 (0.89,85.75)	MBCT									
7.80 (0.56,108.99)	0.89 (0.22,3.67)	CBT+ADM								
10.22 (0.88,118.16)	1.17 (0.42,3.24)	1.31 (0.25,6.95)	IPT							
12.17 (1.00,147.81)	1.39 (0.45,4.32)	1.56 (0.27,8.89)	1.19 (0.28,5.00)	CBT						
12.60 (1.03,153.67)	1.44 (0.46,4.51)	1.62 (0.28,9.25)	1.23 (0.29,5.21)	1.04 (0.23,4.75)	MBCT+ADM					
14.32 (1.17,175.09)	1.64 (0.52,5.16)	1.84 (0.32,10.56)	1.40 (0.33,5.95)	1.18 (0.40,3.50)	1.14 (0.24,5.28)	BA				
0.36 (0.06,2.24)	0.78 (0.23,2.70)	3.73 (0.60,23.19)	1.82 (0.40,8.21)	0.11 (0.01,0.84)	1.29 (0.44,3.78)	1.10 (0.37,3.27)	ADM			
20.20 (1.61,253.75)	2.31 (0.69,7.71)	2.59 (0.43,15.48)	1.98 (0.44,8.79)	1.66 (1.10,2.51)	1.60 (0.33,7.77)	1.41 (0.44,4.52)	0.65 (0.25,1.68)	TAU		
32.45 (2.58,407.80)	3.71 (1.11,12.39)	4.16 (0.70,24.87)	3.17 (0.71,14.13)	2.67 (0.84,8.42)	2.58 (0.53,12.49)	2.27 (0.71,7.27)	0.91 (0.22,3.83)	1.61 (0.47,5.45)	PLA	
66.90 (4.71,950.44)	7.65 (1.80,32.47)	8.58 (1.21,60.77)	6.54 (1.20,35.56)	5.50 (2.24,13.50)	5.31 (0.91,31.15)	4.67 (1.14,19.18)	0.49 (0.13,1.95)	3.31 (1.23,8.90)	2.06 (0.48,8.87)	SUP

eTable 5D: League Table for 12-month follow-up timepoint

CBT+ADM										
1.15 (0.63,2.09)	SUP+ADM									
1.59 (0.27,9.25)	1.38 (0.21,8.88)	CBT								
1.78 (0.42,7.59)	1.55 (0.32,7.43)	1.12 (0.37,3.43)	MBCT							
2.06 (0.37,11.53)	1.79 (0.29,11.09)	1.30 (0.30,5.55)	1.16 (0.41,3.30)	MBCT+ADM						
2.11 (0.42,10.72)	1.83 (0.32,10.37)	1.33 (0.35,5.07)	1.18 (0.49,2.86)	1.02 (0.28,3.69)	IPT					
2.54 (0.44,14.80)	2.21 (0.34,14.21)	1.60 (0.55,4.68)	1.42 (0.47,4.35)	1.23 (0.29,5.26)	1.20 (0.32,4.58)	BA				
2.48 (0.41,15.10)	2.16 (0.32,14.46)	1.56 (1.06,2.31)	1.39 (0.43,4.54)	1.20 (0.27,5.41)	1.18 (0.29,4.74)	0.98 (0.31,3.06)	TAU			
4.76 (0.74,30.59)	0.57 (0.12,2.74)	0.12 (0.02,0.73)	0.84 (0.25,2.82)	1.73 (0.60,5.01)	1.82 (0.43,7.68)	1.08 (0.37,3.13)	1.30 (0.58,2.94)	ADM		
4.23 (0.69,25.88)	3.68 (0.55,24.78)	2.67 (0.84,8.42)	2.37 (0.72,7.80)	2.05 (0.45,9.28)	2.01 (0.49,8.13)	1.67 (0.53,5.27)	1.71 (0.51,5.75)	1.29 (0.30,5.56)	PLA	
7.14 (1.03,49.48)	6.21 (0.82,47.10)	4.50 (2.03,10.00)	4.01 (1.02,15.80)	3.46 (0.66,18.16)	3.39 (0.71,16.07)	2.81 (0.74,10.72)	2.88 (1.18,7.00)	0.36 (0.09,1.49)	1.69 (0.42,6.85)	SUP

eTable 5E: League Table for 15-month follow-up timepoint

CBT										
1.20 (0.42,3.44)	BA									
1.54 (1.05,2.26)	1.28 (0.42,3.94)	TAU								
1.99 (0.63,6.35)	1.66 (0.51,5.39)	1.29 (0.38,4.38)	CBT+ADM							
2.28 (0.62,8.37)	1.90 (0.51,7.09)	1.48 (0.38,5.74)	1.14 (0.64,2.06)	SUP+ADM						
2.52 (0.80,7.96)	2.10 (0.65,6.76)	1.64 (0.49,5.50)	1.26 (0.76,2.09)	1.11 (0.51,2.40)	MBCT					
2.90 (0.75,11.17)	2.41 (0.62,9.46)	1.88 (0.46,7.64)	1.45 (0.61,3.46)	1.27 (0.44,3.62)	1.15 (0.49,2.70)	IPT				
3.07 (0.80,11.77)	2.56 (0.66,9.97)	1.99 (0.49,8.06)	1.54 (0.65,3.64)	1.34 (0.47,3.81)	1.22 (0.52,2.84)	1.06 (0.48,2.32)	PLA			
3.25 (0.75,14.04)	2.71 (0.62,11.87)	2.11 (0.46,9.57)	1.63 (0.58,4.59)	1.42 (0.43,4.68)	1.29 (0.46,3.59)	1.12 (0.32,3.90)	1.06 (0.31,3.66)	MBCT+ADM		
3.81 (1.86,7.80)	3.18 (0.89,11.36)	2.47 (1.10,5.57)	1.91 (0.49,7.47)	1.67 (0.38,7.37)	1.51 (0.39,5.86)	1.32 (0.29,6.07)	1.24 (0.27,5.71)	1.17 (0.23,5.99)	SUP	
0.12 (0.02,0.64)	3.12 (1.02,9.55)	1.49 (1.06,2.09)	0.58 (0.14,2.34)	0.92 (0.30,2.83)	3.75 (1.25,11.24)	0.38 (0.10,1.37)	1.41 (0.29,6.75)	1.88 (1.30,2.72)	1.29 (0.59,2.84)	ADM

eTable 5F: League Table for 18-month follow-up timepoint

CBT									
1.21 (0.43,3.45)	BA								
1.45 (0.99,2.12)	1.20 (0.39,3.64)	TAU							
2.22 (0.69,7.18)	1.83 (0.55,6.04)	1.53 (0.45,5.25)	CBT+ADM						
2.43 (0.65,9.01)	2.00 (0.53,7.56)	1.67 (0.43,6.54)	1.09 (0.61,1.96)	SUP+ADM					
2.57 (0.67,9.87)	2.11 (0.54,8.28)	1.77 (0.44,7.16)	1.16 (0.51,2.65)	1.06 (0.38,2.91)	PLA				
2.63 (0.68,10.12)	2.17 (0.55,8.48)	1.81 (0.45,7.34)	1.18 (0.52,2.71)	1.08 (0.39,2.99)	1.02 (0.48,2.18)	IPT			
2.95 (0.90,9.63)	2.43 (0.73,8.09)	2.03 (0.59,7.03)	1.33 (0.79,2.24)	1.22 (0.56,2.66)	1.15 (0.50,2.66)	1.12 (0.48,2.60)	MBCT		
3.59 (1.78,7.27)	2.96 (0.84,10.43)	2.47 (1.11,5.50)	1.62 (0.41,6.36)	1.48 (0.33,6.56)	1.40 (0.31,6.39)	1.37 (0.30,6.25)	1.22 (0.31,4.83)	SUP	
0.13 (0.03,0.69)	3.23 (1.03,10.11)	1.03 (0.33,3.21)	0.34 (0.09,1.29)	1.49 (0.70,3.16)	1.77 (1.24,2.52)	0.30 (0.08,1.09)	3.92 (1.28,12.01)	1.43 (0.30,6.74)	ADM

eTable 5G: League Table for 21-month follow-up timepoint

CBT								
1.43 (0.98,2.08)	TAU							
1.55 (0.74,3.25)	1.09 (0.47,2.50)	IPT						
1.63 (0.57,4.66)	1.15 (0.38,3.49)	1.05 (0.29,3.80)	BA					
3.07 (1.53,6.14)	2.15 (0.98,4.74)	1.98 (0.72,5.45)	1.88 (0.53,6.60)	SUP+ADM				
4.70 (1.25,17.72)	3.29 (0.83,13.09)	3.03 (0.66,13.84)	2.87 (0.75,11.07)	1.53 (0.34,6.85)	CBT+ADM			
6.01 (1.58,22.90)	4.22 (1.05,16.92)	3.88 (0.84,17.86)	3.68 (0.95,14.30)	1.96 (0.43,8.84)	1.28 (0.76,2.15)	MBCT		
7.32 (1.72,31.04)	5.13 (1.15,22.84)	4.72 (0.93,23.91)	4.48 (1.04,19.36)	2.38 (0.48,11.85)	1.56 (0.73,3.31)	1.22 (0.56,2.63)	PLA	
0.08 (0.01,0.48)	1.07 (0.55,2.09)	7.84 (2.18,28.22)	4.80 (1.30,17.64)	1.30 (0.89,1.91)	0.10 (0.02,0.41)	1.67 (1.18,2.36)	5.05 (1.15,22.18)	ADM

eTable 5H: League Table for 24-month follow-up timepoint

CBT								
1.34 (0.82,2.19)	TAU							
1.43 (0.46,4.42)	1.06 (0.31,3.65)	BA						
1.43 (0.62,3.33)	1.07 (0.40,2.84)	1.00 (0.25,4.11)	IPT					
3.71 (0.90,15.25)	2.77 (0.62,12.37)	2.60 (0.62,10.91)	2.59 (0.50,13.41)	CBT+ADM				
3.09 (1.35,7.08)	2.30 (0.89,5.97)	2.16 (0.53,8.79)	2.16 (0.66,7.03)	0.83 (0.16,4.32)	SUP+ADM			
5.57 (1.28,24.13)	4.15 (0.88,19.49)	3.90 (0.88,17.25)	3.88 (0.71,21.06)	1.50 (0.74,3.05)	1.80 (0.33,9.71)	MBCT		
0.13 (0.03,0.62)	0.41 (0.06,2.88)	4.80 (1.22,18.91)	1.84 (1.21,2.80)	6.85 (1.78,26.40)	0.70 (0.32,1.52)	4.78 (0.97,23.44)	ADM	
9.84 (2.07,46.74)	7.33 (1.43,37.59)	6.89 (1.42,33.39)	6.86 (1.17,40.34)	2.65 (1.09,6.42)	3.18 (0.54,18.59)	1.77 (0.67,4.66)	0.81 (0.46,1.44)	PLA

eFigure 4A: Funnel plot of primary outcome in 9-month follow up (with CBT)

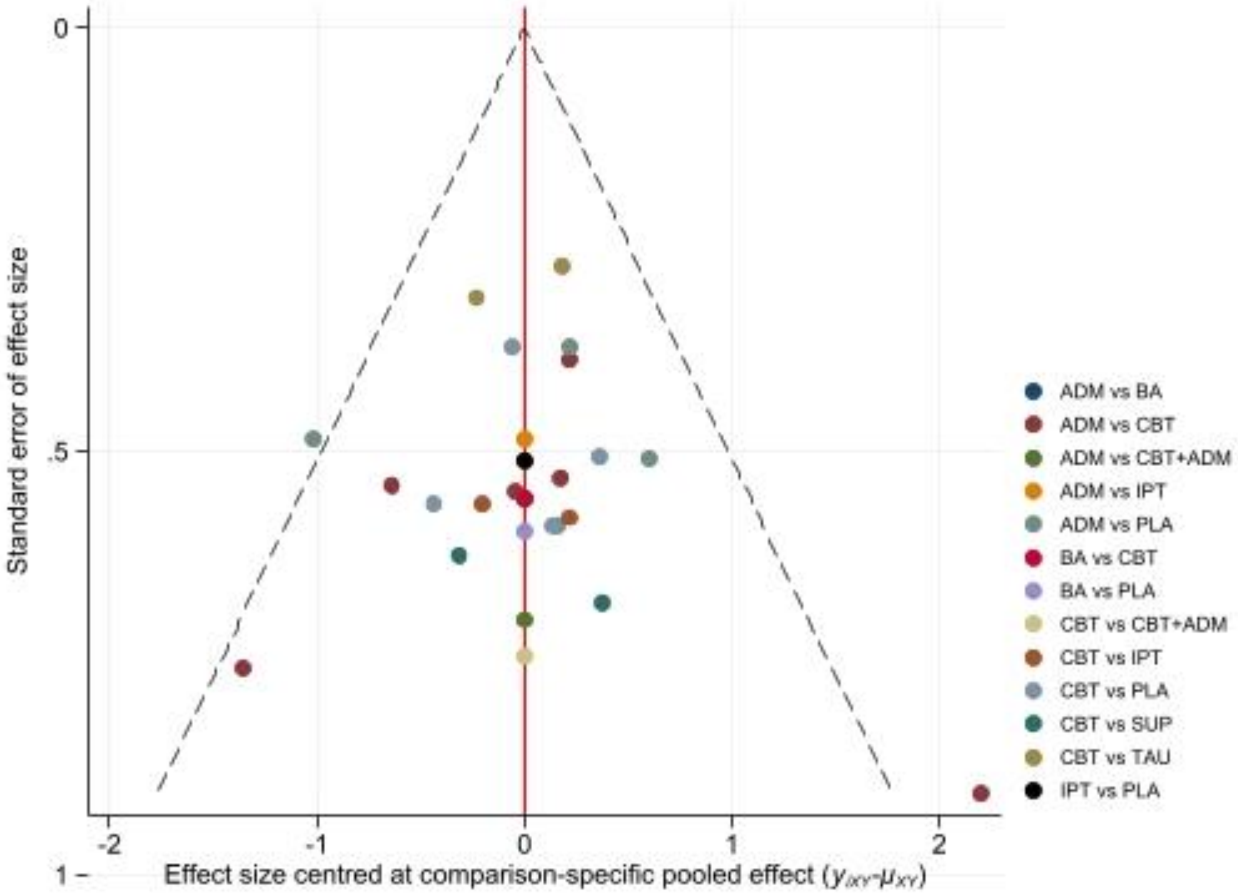

eFigure 4B: Funnel plot of primary outcome in 9-month follow up (without CBT)

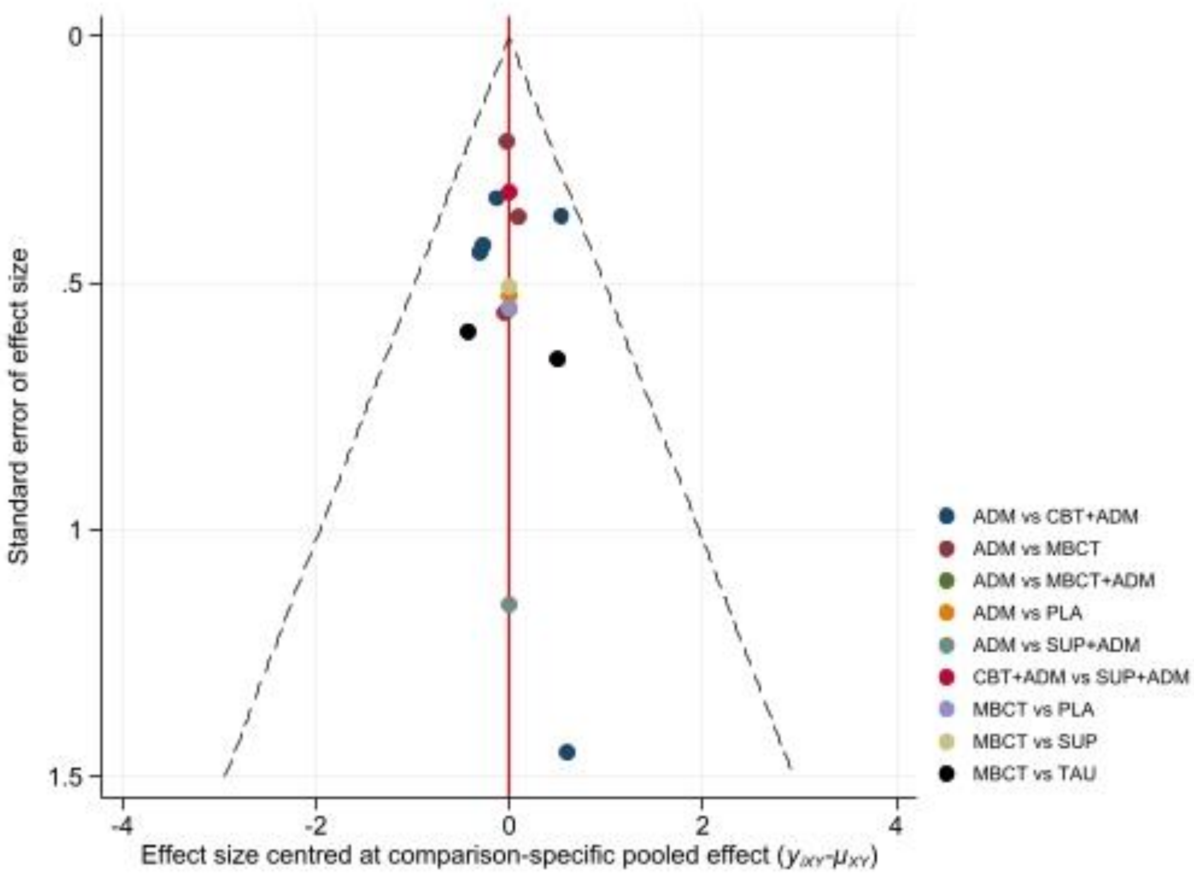

eFigure 5A: Transitivity assessment of sample size

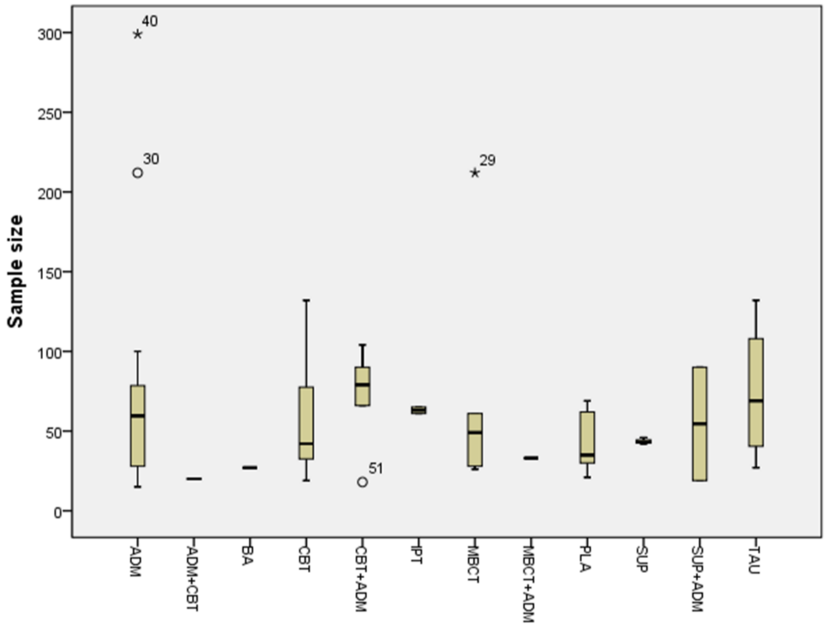

eFigure 5B: Transitivity assessment of mean age

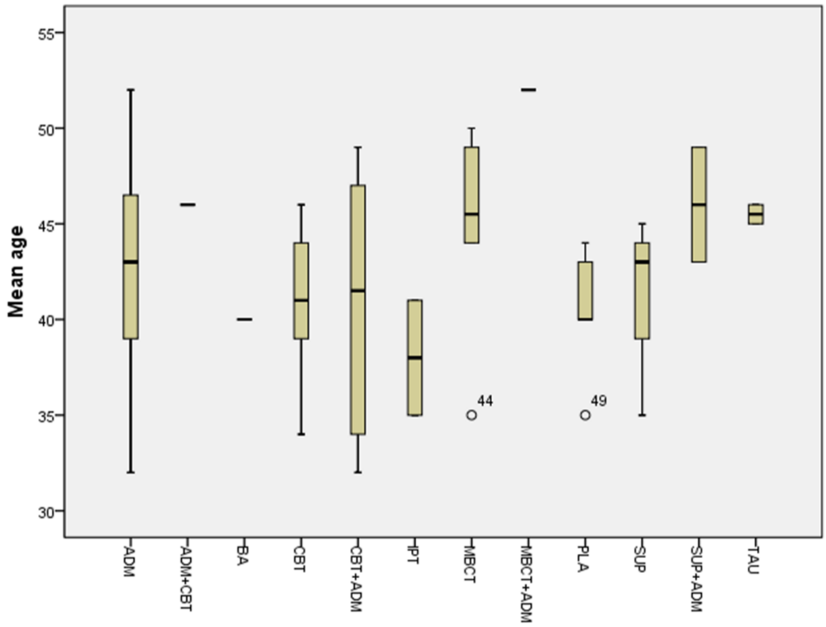

eFigure 5C: Transitivity assessment of public year

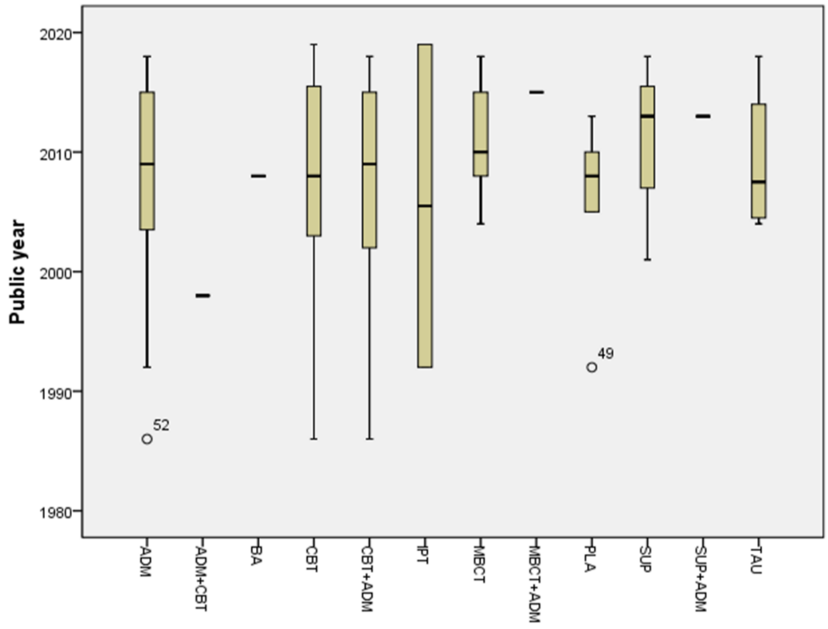

eFigure 5D: Transitivity assessment of women(%)

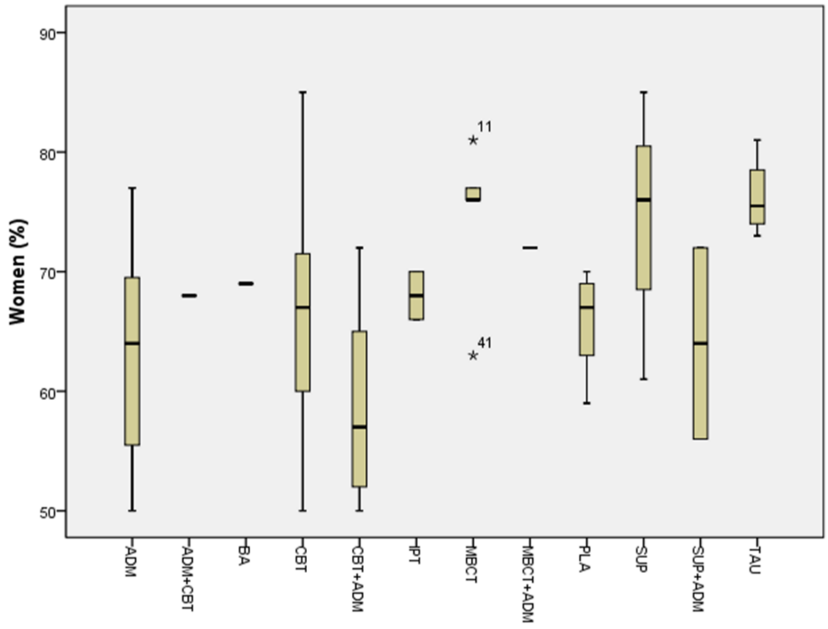

eTable 6A: Inconsistency Test Based on Loop-Specific Approach

Loop	ROR	z-value	P-value	95%CI	tau2
C-F-I-K	2.095	3.765	0	(1.43,3.08)	0
A-D-J	1.669	1.829	0.067	(1.00,2.89)	0.001
A-C-F-I	1.567	2.488	0.013	(1.10,2.23)	0.003
C-F-H-K	1.475	1.281	0.2	(1.00,2.67)	0.006
A-C-D	1.436	1.528	0.126	(1.00,2.29)	0.002
C-F-H-I	1.422	1.061	0.289	(1.00,2.73)	0.011
B-C-H	1.376	0.897	0.37	(1.00,2.77)	0
A-E-H	1.366	2.209	0.027	(1.04,1.80)	0
A-C-F-K	1.346	1.773	0.076	(1.00,1.87)	0.002
A-B-H	1.251	0.487	0.626	(1.00,3.08)	0.033
C-E-H	1.219	1.606	0.108	(1.00,1.55)	0
A-C-E	1.179	1.107	0.268	(1.00,1.58)	0.004
A-F-H	1.094	0.297	0.766	(1.00,1.98)	0.017
A-B-C	1.067	0.17	0.865	(1.00,2.26)	0.014
A-C-H	1.053	0.203	0.839	(1.00,1.74)	0.033

eTable 6B: Inconsistency Test Based on Loop-Specific Approach (continued)

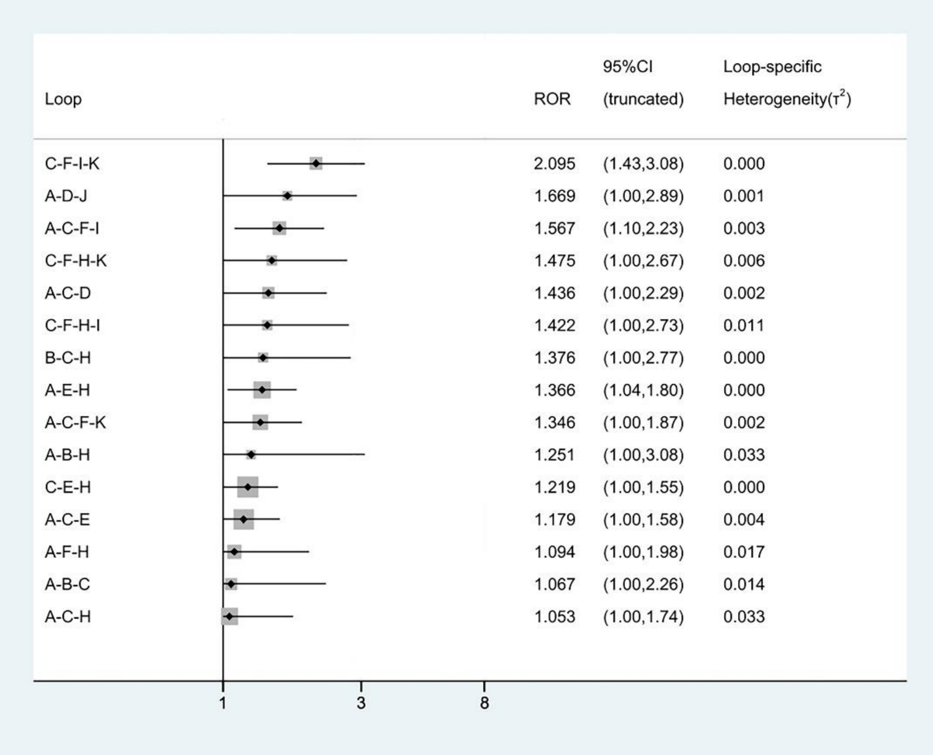

eTable 6C: Evaluation of the incoherence by node-splitting model for inconsistency check in the network meta-analysis for relapse at 9-month follow-up timepoint outcomes

comparison		Direct		Indirect		Difference			
		Coef.	Std.Err.	Coef.	Std.Err.	Coef.	Std.Err.	P>|z|	tau
A:B	*	0.09531	0.697292	0.409253	1.10864	-0.31394	1.302734	0.81	0.421497
A:C	*	0.231141	0.294519	0.474729	0.525134	-0.24359	0.60308	0.686	0.432559
A:D		0.773981	0.298417	0.818213	1.020586	-0.04423	1.064891	0.967	0.410468
A:E		0.446662	0.56566	-0.94299	0.590935	1.389653	0.808409	0.086	0.290753
A:F		0.587444	0.323489	0.705193	0.56129	-0.11775	0.646516	0.855	0.427676
A:G		.	.	.	.	.	.	.	.
A:H	*	-0.4488	0.290549	-0.59862	0.90026	0.149823	0.946128	0.874	0.426492
A:J		2.75684	1.205432	0.533669	0.553751	2.223171	1.326539	0.094	0.360074
B:C	*	0.162519	0.697879	-0.04164	1.123612	0.204157	1.316013	0.877	0.42215
B:H	*	-0.81831	0.725354	-0.23893	1.139478	-0.57938	1.376877	0.674	0.415777
C:D		-0.82048	0.821221	0.781152	0.389576	-1.60164	0.910648	0.079	0.356515
C:E	*	-0.63079	0.496676	-0.01445	1.071287	-0.61634	1.204344	0.609	0.410722
C:H	*	-0.83886	0.322081	-0.42206	0.635328	-0.4168	0.71453	0.56	0.408796
C:I		-1.70687	0.480356	0.32584	0.610492	-2.03271	0.77725	**0.009**	0.201564
C:K		-0.50251	0.230514	-2.25501	0.536507	1.752503	0.587395	**0.003**	0.129779
D:J		-0.14887	0.478562	2.074009	1.237168	-2.22288	1.326502	0.094	0.360075
E:H		0.019442	0.653195	-0.52043	0.6824	0.539874	0.944524	0.568	0.407851
F:H		-0.62289	0.681577	-1.23759	0.398485	0.614699	0.789485	0.436	0.402628
F:I		-0.25624	0.546122	-2.28891	0.553052	2.032667	0.777249	**0.009**	0.201569
F:K		-2.21535	0.450811	-0.46275	0.373812	-1.75259	0.587423	**0.003**	0.12981

Note

1. A=ADM; B=BA; C=CBT; D=CBT+ADM; E=IPT; F=MBCT; G=MBCT+ADM; H=PLA; I=SUP; J=SUP+ADM; K=TAU
2. Coef. = the standardized mean difference between sides
3. Std. Err. = standard error
4. p-value for the difference between direct and indirect comparisons

* Warning: all the evidence about these contrasts comes from the trials which directly compare them.

eTable 7A: Sensitive analyses: League Table for 3-month follow-up timepoint when limit to studies reporting the narrowly defined diagnosis

SUP+ADM									
2.86 (0.07,119.46)	MBCT+ADM								
8.61 (0.41,183.03)	3.01 (0.31,29.00)	MBCT							
13.93 (0.54,356.71)	4.87 (0.40,59.98)	1.62 (0.44,6.00)	CBT						
12.72 (0.46,353.35)	4.44 (0.32,60.79)	1.48 (0.33,6.63)	0.91 (0.14,5.82)	CBT+ADM					
16.02 (0.62,411.82)	5.60 (0.45,69.32)	1.86 (0.50,6.97)	1.15 (0.34,3.84)	1.26 (0.20,8.09)	BA				
0.51 (0.13,2.05)	1.10 (0.33,3.65)	1.04 (0.23,4.65)	0.10 (0.00,3.02)	4.66 (0.54,39.93)	0.95 (0.28,3.21)	ADM			
31.55 (1.18,845.07)	11.02 (0.84,143.94)	3.66 (0.89,15.15)	2.26 (1.31,3.90)	2.48 (0.36,17.10)	1.97 (0.52,7.40)	0.56 (0.33,0.95)	TAU		
34.34 (1.33,883.31)	12.00 (0.97,148.72)	3.99 (1.06,14.97)	2.46 (0.74,8.26)	2.70 (0.42,17.36)	2.14 (0.63,7.26)	0.65 (0.15,2.94)	1.09 (0.29,4.10)	PLA	
75.99 (2.04,2825.70)	26.56 (1.35,521.50)	8.82 (1.11,69.83)	5.45 (1.10,27.01)	5.98 (0.52,69.09)	4.74 (0.64,35.19)	0.83 (0.20,3.41)	2.41 (0.44,13.05)	2.21 (0.30,16.45)	SUP

eTable 7B: Sensitive analyses: League Table for 6-month follow-up timepoint when limit to studies reporting the narrowly defined diagnosis

SUP+ADM									
8.74 (0.79,96.16)	MBCT								
10.13 (0.64,160.94)	1.16 (0.23,5.81)	CBT+ADM							
11.94 (0.85,168.07)	1.37 (0.34,5.51)	1.18 (0.17,8.38)	BA						
11.25 (0.75,167.91)	1.29 (0.29,5.78)	1.11 (0.14,8.54)	0.94 (0.15,6.12)	MBCT+ADM					
14.17 (1.03,195.58)	1.62 (0.42,6.30)	1.40 (0.20,9.68)	1.19 (0.34,4.16)	1.26 (0.20,7.96)	CBT				
0.84 (0.20,3.46)	0.96 (0.22,4.20)	2.62 (0.36,19.38)	1.32 (0.37,4.67)	1.11 (0.33,3.76)	0.57 (0.04,9.36)	ADM			
24.36 (1.65,359.64)	2.79 (0.63,12.28)	2.41 (0.32,18.22)	2.04 (0.51,8.20)	2.16 (0.31,15.04)	1.72 (0.94,3.13)	0.55 (0.31,1.00)	TAU		
37.80 (2.65,538.73)	4.32 (1.05,17.86)	3.73 (0.52,26.97)	3.17 (0.85,11.87)	3.36 (0.51,22.21)	2.67 (0.74,9.59)	0.78 (0.16,3.85)	1.55 (0.38,6.38)	PLA	
113.38 (5.06,2540.60)	12.97 (1.51,111.30)	11.20 (0.87,143.91)	9.50 (1.18,76.54)	10.08 (0.84,120.97)	8.00 (1.51,42.38)	0.64 (0.14,2.88)	4.66 (0.79,27.39)	3.00 (0.37,24.55)	SUP

eTable 7C: Sensitive analyses: League Table for 9-month follow-up timepoint when limit to studies reporting the narrowly defined diagnosis

SUP+ADM										
8.74 (0.89,85.75)	MBCT									
7.80 (0.56,108.99)	0.89 (0.22,3.67)	CBT+ADM								
12.17 (1.00,147.81)	1.39 (0.45,4.32)	1.56 (0.27,8.89)	CBT							
12.60 (1.03,153.67)	1.44 (0.46,4.51)	1.62 (0.28,9.25)	1.04 (0.23,4.75)	MBCT+ADM						
14.32 (1.17,175.09)	1.64 (0.52,5.16)	1.84 (0.32,10.56)	1.18 (0.40,3.50)	1.14 (0.24,5.28)	BA					
1.05 (0.33,3.32)	1.29 (0.44,3.78)	3.73 (0.60,23.19)	0.11 (0.01,0.84)	1.12 (0.27,4.70)	1.10 (0.37,3.27)	ADM				
20.20 (1.61,253.75)	2.31 (0.69,7.71)	2.59 (0.43,15.48)	1.66 (1.10,2.51)	1.60 (0.33,7.77)	1.41 (0.44,4.52)	0.56 (0.39,0.80)	TAU			
28.32 (1.83,439.38)	3.24 (0.65,16.06)	3.63 (0.46,28.94)	2.33 (0.75,7.22)	2.25 (0.34,15.01)	1.98 (0.41,9.52)	1.28 (0.37,4.40)	1.40 (0.42,4.68)	IPT		
32.45 (2.58,407.80)	3.71 (1.11,12.39)	4.16 (0.70,24.87)	2.67 (0.84,8.42)	2.58 (0.53,12.49)	2.27 (0.71,7.27)	0.49 (0.13,1.95)	1.61 (0.47,5.45)	1.15 (0.23,5.76)	PLA	
66.90 (4.71,950.44)	7.65 (1.80,32.47)	8.58 (1.21,60.77)	5.50 (2.24,13.50)	5.31 (0.91,31.15)	4.67 (1.14,19.18)	1.80 (0.38,8.56)	3.31 (1.23,8.90)	2.36 (0.56,10.03)	2.06 (0.48,8.87)	SUP

eTable 7D: Sensitive analyses: League Table for 12-month follow-up timepoint when limit to studies reporting the narrowly defined diagnosis

CBT+ADM										
1.15 (0.63,2.09)	SUP+ADM									
1.59 (0.27,9.25)	1.38 (0.21,8.88)	CBT								
1.78 (0.42,7.59)	1.55 (0.32,7.43)	1.12 (0.37,3.43)	MBCT							
2.06 (0.37,11.53)	1.79 (0.29,11.09)	1.30 (0.30,5.55)	1.16 (0.41,3.30)	MBCT+ADM						
2.48 (0.41,15.10)	2.16 (0.32,14.46)	1.56 (1.06,2.31)	1.39 (0.43,4.54)	1.20 (0.27,5.41)	TAU					
2.54 (0.44,14.80)	2.21 (0.34,14.21)	1.60 (0.55,4.68)	1.42 (0.47,4.35)	1.23 (0.29,5.26)	1.02 (0.33,3.21)	BA				
4.76 (0.74,30.59)	0.88 (0.29,2.73)	0.12 (0.02,0.73)	1.73 (0.60,5.01)	0.78 (0.18,3.35)	1.54 (1.09,2.18)	1.08 (0.37,3.13)	ADM			
3.72 (0.53,26.13)	3.23 (0.42,24.85)	2.34 (1.02,5.39)	2.09 (0.52,8.39)	1.80 (0.34,9.61)	1.50 (0.60,3.76)	1.46 (0.38,5.70)	1.19 (0.35,3.98)	IPT		
4.23 (0.69,25.88)	3.68 (0.55,24.78)	2.67 (0.84,8.42)	2.37 (0.72,7.80)	2.05 (0.45,9.28)	1.71 (0.51,5.75)	1.67 (0.53,5.27)	0.36 (0.09,1.49)	1.14 (0.28,4.71)	PLA	
7.14 (1.03,49.48)	6.21 (0.82,47.10)	4.50 (2.03,10.00)	4.01 (1.02,15.80)	3.46 (0.66,18.16)	2.88 (1.18,7.00)	2.81 (0.74,10.72)	1.35 (0.35,5.21)	1.92 (0.61,6.09)	1.69 (0.42,6.85)	SUP

eTable 7E: Sensitive analyses: League Table for 15-month follow-up timepoint when limit to studies reporting the narrowly defined diagnosis

CBT										
1.20 (0.42,3.44)	BA									
1.54 (1.05,2.26)	1.28 (0.42,3.94)	TAU								
1.98 (0.62,6.33)	1.65 (0.51,5.37)	1.29 (0.38,4.37)	CBT+ADM							
2.06 (0.93,4.56)	1.71 (0.46,6.42)	1.33 (0.55,3.23)	1.04 (0.25,4.24)	IPT						
2.27 (0.62,8.33)	1.89 (0.51,7.06)	1.47 (0.38,5.71)	1.14 (0.64,2.06)	1.10 (0.24,5.07)	SUP+ADM					
2.52 (0.80,7.96)	2.10 (0.65,6.76)	1.64 (0.49,5.50)	1.27 (0.77,2.12)	1.23 (0.30,4.97)	1.11 (0.51,2.42)	MBCT				
3.25 (0.75,14.04)	2.71 (0.62,11.87)	2.11 (0.46,9.57)	1.64 (0.58,4.63)	1.58 (0.30,8.36)	1.43 (0.43,4.73)	1.29 (0.46,3.59)	MBCT+ADM			
3.81 (1.86,7.80)	3.18 (0.89,11.36)	2.47 (1.10,5.57)	1.92 (0.49,7.53)	1.85 (0.64,5.41)	1.68 (0.38,7.43)	1.51 (0.39,5.86)	1.17 (0.23,5.99)	SUP		
0.12 (0.02,0.64)	3.12 (1.02,9.55)	0.67 (0.19,2.35)	0.38 (0.10,1.37)	3.75 (1.25,11.24)	1.15 (0.44,3.04)	1.89 (1.30,2.76)	1.82 (0.47,7.08)	1.49 (1.06,2.09)	ADM	
4.37 (1.21,15.84)	3.64 (0.99,13.42)	2.83 (0.74,10.86)	2.21 (1.02,4.77)	2.13 (0.47,9.66)	1.93 (0.73,5.08)	1.73 (0.81,3.68)	1.34 (0.41,4.37)	1.15 (0.26,5.00)	1.09 (0.35,3.36)	PLA

eTable 7F: Sensitive analyses: League Table for 18-month follow-up timepoint when limit to studies reporting the narrowly defined diagnosis

CBT									
1.21 (0.43,3.45)	BA								
1.45 (0.99,2.12)	1.20 (0.39,3.64)	TAU							
1.84 (0.85,3.97)	1.52 (0.41,5.55)	1.27 (0.54,2.99)	IPT						
2.24 (0.69,7.27)	1.85 (0.56,6.11)	1.54 (0.45,5.31)	1.22 (0.30,4.96)	CBT+ADM					
2.45 (0.66,9.11)	2.02 (0.53,7.65)	1.69 (0.43,6.62)	1.33 (0.29,6.09)	1.09 (0.61,1.96)	SUP+ADM				
2.95 (0.90,9.63)	2.43 (0.73,8.09)	2.03 (0.59,7.03)	1.60 (0.39,6.56)	1.32 (0.78,2.23)	1.20 (0.55,2.64)	MBCT			
3.59 (1.78,7.27)	2.96 (0.84,10.43)	2.47 (1.11,5.50)	1.95 (0.69,5.53)	1.60 (0.41,6.31)	1.47 (0.33,6.51)	1.22 (0.31,4.83)	SUP		
4.11 (1.12,15.13)	3.39 (0.90,12.70)	2.83 (0.73,10.99)	2.23 (0.49,10.13)	1.83 (0.86,3.92)	1.68 (0.64,4.37)	1.39 (0.65,3.01)	1.14 (0.26,5.03)	PLA	
0.13 (0.03,0.69)	3.23 (1.03,10.11)	0.52 (0.15,1.84)	3.92 (1.28,12.01)	0.30 (0.08,1.09)	1.33 (0.91,1.95)	1.75 (1.22,2.51)	1.03 (0.33,3.21)	2.13 (0.55,8.28)	ADM

eTable 7G: Sensitive analyses: League Table for 21-month follow-up timepoint when limit to studies reporting the narrowly defined diagnosis

CBT								
1.43 (0.98,2.08)	TAU							
1.55 (0.74,3.25)	1.09 (0.47,2.50)	IPT						
1.63 (0.57,4.66)	1.15 (0.38,3.49)	1.05 (0.29,3.80)	BA					
3.07 (1.53,6.14)	2.15 (0.98,4.74)	1.98 (0.72,5.45)	1.88 (0.53,6.60)	SUP				
4.73 (1.25,17.87)	3.31 (0.83,13.21)	3.05 (0.67,13.95)	2.89 (0.75,11.16)	1.54 (0.34,6.91)	CBT+ADM			
6.01 (1.58,22.90)	4.22 (1.05,16.92)	3.88 (0.84,17.86)	3.68 (0.95,14.30)	1.96 (0.43,8.84)	1.27 (0.75,2.15)	MBCT		
7.32 (1.72,31.04)	5.13 (1.15,22.84)	4.72 (0.93,23.91)	4.48 (1.04,19.36)	2.38 (0.48,11.85)	1.55 (0.73,3.30)	1.22 (0.56,2.63)	PLA	
0.08 (0.01,0.48)	1.07 (0.55,2.09)	7.84 (2.18,28.22)	4.80 (1.30,17.64)	1.30 (0.89,1.91)	0.10 (0.02,0.41)	1.66 (1.16,2.37)	5.05 (1.15,22.18)	ADM

eTable 7H: Sensitive analyses: League Table for 24-month follow-up timepoint when limit to studies reporting the narrowly defined diagnosis

CBT								
1.34 (0.74,2.44)	TAU							
1.43 (0.42,4.86)	1.06 (0.27,4.15)	BA						
1.43 (0.55,3.77)	1.07 (0.34,3.32)	1.00 (0.21,4.78)	IPT					
3.79 (0.83,17.29)	2.82 (0.55,14.39)	2.65 (0.57,12.35)	2.64 (0.44,15.97)	CBT+ADM				
3.20 (1.33,7.71)	2.38 (0.83,6.84)	2.24 (0.50,10.12)	2.23 (0.60,8.24)	0.84 (0.15,4.88)	SUP			
5.57 (1.11,27.90)	4.14 (0.74,23.08)	3.90 (0.76,19.92)	3.88 (0.59,25.43)	1.47 (0.60,3.63)	1.74 (0.28,10.91)	MBCT		
0.13 (0.02,0.71)	0.42 (0.05,3.60)	4.80 (1.12,20.48)	1.81 (1.09,3.01)	6.85 (1.64,28.61)	0.70 (0.28,1.74)	4.78 (0.85,26.83)	ADM	
9.84 (1.80,53.64)	7.31 (1.21,44.15)	6.89 (1.24,38.26)	6.86 (0.97,48.32)	2.60 (0.91,7.39)	3.07 (0.45,20.77)	1.77 (0.54,5.74)	0.81 (0.39,1.71)	PLA

eTable 8A: Sensitive analyses: League Table for 3-month follow-up timepoint when limit to studies reporting the narrowly defined relapse

SUP+ADM							
8.33 (0.39,176.93)	CBT+ADM						
8.61 (0.41,183.03)	1.03 (0.49,2.16)	MBCT					
0.21 (0.07,0.68)	1.83 (1.09,3.09)	0.51 (0.13,2.05)	ADM				
36.56 (1.42,941.23)	4.39 (1.17,16.51)	4.24 (1.13,15.97)	2.40 (0.71,8.10)	CBT			
72.61 (2.87,1837.35)	8.72 (2.42,31.41)	8.43 (2.34,30.39)	0.56 (0.33,0.95)	1.99 (0.79,4.97)	PLA		
82.77 (3.07,2229.67)	9.94 (2.37,41.62)	9.61 (2.29,40.26)	0.04 (0.00,0.54)	2.26 (1.31,3.90)	1.14 (0.39,3.31)	TAU	
199.39 (5.34,7451.40)	23.94 (3.00,191.11)	23.15 (2.90,184.82)	0.33 (0.06,1.76)	5.45 (1.10,27.01)	2.75 (0.43,17.36)	2.41 (0.44,13.05)	SUP

eTable 8B: Sensitive analyses: League Table for 6-month follow-up timepoint when limit to studies reporting the narrowly defined relapse

SUP+ADM							
8.22 (0.81,83.15)	CBT+ADM						
8.46 (0.83,85.89)	1.03 (0.51,2.07)	MBCT					
0.35 (0.14,0.83)	1.92 (1.21,3.03)	0.81 (0.23,2.91)	ADM				
25.29 (2.21,289.83)	3.08 (1.12,8.43)	2.99 (1.08,8.27)	1.61 (0.65,3.94)	CBT			
43.47 (3.62,522.21)	5.29 (1.73,16.16)	5.14 (1.67,15.84)	0.05 (0.01,0.44)	1.72 (1.06,2.78)	TAU		
45.48 (3.99,518.01)	5.53 (2.05,14.93)	5.38 (1.98,14.64)	0.54 (0.33,0.87)	1.80 (0.80,4.03)	1.05 (0.41,2.68)	PLA	
202.26 (11.01,3716.46)	24.61 (3.75,161.53)	23.91 (3.63,157.75)	0.42 (0.10,1.69)	8.00 (1.63,39.19)	4.65 (0.88,24.48)	4.45 (0.75,26.41)	SUP

eTable 8C: Sensitive analyses: League Table for 9-month follow-up timepoint when limit to studies reporting the narrowly defined relapse

SUP+ADM							
6.97 (0.70,69.32)	CBT+ADM						
8.74 (0.89,85.75)	1.25 (0.71,2.21)	MBCT					
15.75 (1.45,170.50)	2.26 (0.93,5.46)	1.80 (0.77,4.21)	CBT				
0.46 (0.22,0.96)	2.26 (1.46,3.50)	1.05 (0.33,3.32)	1.00 (0.46,2.16)	ADM			
26.14 (2.33,293.17)	3.75 (1.41,9.94)	2.99 (1.16,7.68)	1.66 (1.10,2.51)	0.13 (0.03,0.64)	TAU		
34.45 (3.21,369.51)	4.94 (2.09,11.65)	3.94 (1.73,8.96)	2.19 (1.05,4.58)	0.56 (0.39,0.80)	1.32 (0.57,3.07)	PLA	
86.57 (6.79,1104.14)	12.42 (3.52,43.78)	9.90 (2.88,34.08)	5.50 (2.24,13.50)	0.46 (0.13,1.62)	3.31 (1.23,8.90)	2.51 (0.79,8.04)	SUP

eTable 8D: Sensitive analyses: League Table for 12-month follow-up timepoint when limit to studies reporting the narrowly defined relapse

CBT+ADM							
1.15 (0.63,2.09)	SUP+ADM						
1.36 (0.60,3.06)	1.18 (0.43,3.24)	CBT					
1.38 (0.82,2.34)	1.20 (0.54,2.66)	1.02 (0.46,2.24)	MBCT				
2.12 (0.86,5.23)	1.85 (0.63,5.45)	1.56 (1.06,2.31)	1.54 (0.64,3.70)	TAU			
2.13 (1.43,3.17)	0.77 (0.39,1.54)	1.57 (0.77,3.19)	0.88 (0.29,2.73)	3.48 (0.79,15.25)	ADM		
2.75 (1.24,6.09)	2.40 (0.89,6.47)	2.03 (0.98,4.21)	1.99 (0.92,4.30)	1.30 (0.57,2.97)	0.65 (0.46,0.91)	PLA	
6.11 (1.96,19.10)	5.32 (1.47,19.25)	4.50 (2.03,10.00)	4.42 (1.44,13.57)	2.88 (1.18,7.00)	0.77 (0.22,2.67)	2.22 (0.75,6.54)	SUP

eTable 8E: Sensitive analyses: League Table for 15-month follow-up timepoint when limit to studies reporting the narrowly defined relapse

CBT+ADM							
1.14 (0.64,2.06)	SUP+ADM						
1.27 (0.77,2.12)	1.11 (0.51,2.42)	MBCT					
1.34 (0.62,2.88)	1.17 (0.44,3.07)	1.05 (0.49,2.23)	CBT				
1.89 (1.30,2.76)	0.86 (0.44,1.68)	0.92 (0.30,2.83)	1.42 (0.72,2.77)	ADM			
2.06 (0.87,4.86)	1.80 (0.64,5.10)	1.62 (0.70,3.76)	1.54 (1.05,2.26)	0.31 (0.08,1.27)	TAU		
2.21 (1.02,4.77)	1.93 (0.73,5.08)	1.73 (0.81,3.68)	1.65 (0.82,3.32)	0.67 (0.48,0.95)	1.07 (0.48,2.37)	PLA	
5.09 (1.78,14.57)	4.45 (1.34,14.84)	4.00 (1.42,11.30)	3.81 (1.86,7.80)	1.48 (0.43,5.15)	2.47 (1.10,5.57)	2.31 (0.85,6.28)	SUP

eTable 8F: Sensitive analyses: League Table for 18-month follow-up timepoint when limit to studies reporting the narrowly defined relapse

CBT+ADM							
1.09 (0.61,1.96)	SUP+ADM						
1.32 (0.78,2.23)	1.20 (0.55,2.64)	MBCT					
1.49 (0.71,3.11)	1.36 (0.53,3.49)	1.13 (0.54,2.39)	CBT				
1.83 (0.86,3.92)	1.68 (0.64,4.37)	1.39 (0.65,3.01)	1.23 (0.63,2.42)	PLA			
1.75 (1.22,2.51)	0.95 (0.49,1.86)	1.03 (0.33,3.21)	1.17 (0.62,2.23)	1.33 (0.91,1.95)	ADM		
2.16 (0.95,4.95)	1.98 (0.72,5.46)	1.65 (0.71,3.80)	1.45 (0.99,2.12)	1.18 (0.54,2.56)	0.31 (0.08,1.23)	TAU	
5.35 (1.93,14.83)	4.90 (1.51,15.85)	4.07 (1.46,11.36)	3.59 (1.78,7.27)	2.92 (1.10,7.75)	1.91 (0.54,6.68)	2.47 (1.11,5.50)	SUP

eTable 8G: Sensitive analyses: League Table for 21-month follow-up timepoint when limit to studies reporting the narrowly defined relapse

CBT+ADM						
1.27 (0.75,2.15)	MBCT					
1.55 (0.73,3.30)	1.22 (0.56,2.63)	PLA				
1.66 (1.16,2.37)	1.30 (0.89,1.91)	1.07 (0.55,2.09)	ADM			
2.12 (1.04,4.34)	1.67 (0.81,3.45)	1.37 (0.71,2.66)	1.28 (0.69,2.38)	CBT		
3.03 (1.35,6.80)	2.38 (1.05,5.40)	1.96 (0.91,4.19)	3.93 (1.55,9.95)	1.43 (0.98,2.08)	TAU	
6.52 (2.41,17.64)	5.12 (1.88,13.99)	4.21 (1.62,10.97)	0.26 (0.07,1.01)	3.07 (1.53,6.14)	2.15 (0.98,4.74)	SUP

eTable 8H: Sensitive analyses: League Table for 24-month follow-up timepoint when limit to studies reporting the narrowly defined relapse

CBT+ADM						
1.47 (0.60,3.63)	MBCT					
1.81 (1.09,3.01)	1.23 (0.58,2.59)	ADM				
2.00 (0.71,5.60)	1.36 (0.43,4.36)	1.11 (0.45,2.70)	CBT			
2.60 (0.91,7.39)	1.77 (0.54,5.74)	1.44 (0.58,3.58)	1.30 (0.52,3.22)	PLA		
2.69 (0.82,8.83)	1.83 (0.50,6.76)	3.54 (1.01,12.39)	1.34 (0.74,2.44)	1.04 (0.35,3.07)	TAU	
6.40 (1.66,24.75)	4.36 (1.01,18.72)	0.31 (0.05,1.92)	3.20 (1.33,7.70)	2.46 (0.70,8.73)	2.38 (0.83,6.84)	SUP

eTable 9A: Sensitive analyses: League Table for 3-month follow-up timepoint when limit to studies in which the population was in remission at the time of grouping

SUP+ADM								
8.61 (0.41,183.03)	MBCT							
9.79 (0.46,208.95)	1.14 (0.53,2.42)	CBT+ADM						
9.16 (0.27,309.95)	1.06 (0.16,7.11)	0.94 (0.14,6.30)	PLA					
14.21 (0.46,438.78)	1.65 (0.29,9.25)	1.45 (0.26,8.20)	1.55 (0.25,9.63)	IPT				
1.77 (1.05,2.99)	1.56 (0.90,2.70)	36.50 (1.07,1241.48)	1.07 (0.21,5.55)	0.11 (0.00,2.67)	ADM			
145.05 (1.75,12037.42)	16.84 (0.64,445.57)	14.82 (0.56,393.68)	15.84 (0.39,649.76)	10.21 (0.27,383.87)	0.03 (0.00,1.72)	CBT		
328.40 (3.83,28177.30)	38.12 (1.38,1055.05)	33.54 (1.21,932.13)	35.86 (0.84,1530.51)	23.11 (0.59,905.07)	2.12 (0.26,17.39)	2.26 (1.31,3.90)	TAU	
791.01 (7.20,86910.73)	91.83 (2.40,3516.75)	80.79 (2.10,3105.87)	86.39 (1.51,4927.85)	55.67 (1.06,2932.52)	1.95 (0.49,7.79)	5.45 (1.10,27.01)	2.41 (0.44,13.05)	SUP

eTable 9B: Sensitive analyses: League Table for 6-month follow-up timepoint when limit to studies in which the population was in remission at the time of grouping

SUP+ADM								
8.34 (0.84,82.49)	MBCT							
9.50 (0.95,94.68)	1.14 (0.62,2.09)	CBT+ADM						
9.87 (0.76,127.44)	1.18 (0.33,4.24)	1.04 (0.29,3.77)	PLA					
14.58 (1.18,180.49)	1.75 (0.53,5.74)	1.53 (0.46,5.11)	1.48 (0.44,4.94)	IPT				
24.68 (0.89,687.69)	2.96 (0.25,35.35)	2.60 (0.22,31.27)	2.50 (0.16,38.32)	1.69 (0.11,24.92)	CBT			
1.89 (1.26,2.84)	1.66 (1.06,2.60)	4.50 (0.30,68.28)	1.08 (0.35,3.30)	0.64 (0.06,7.37)	2.37 (0.15,36.32)	ADM		
42.43 (1.48,1218.42)	5.09 (0.41,63.27)	4.47 (0.36,55.95)	4.30 (0.27,68.33)	2.91 (0.19,44.46)	1.72 (1.10,2.69)	4.45 (0.94,21.12)	TAU	
197.40 (4.98,7818.95)	23.66 (1.26,445.66)	20.78 (1.10,393.70)	20.00 (0.86,466.09)	13.54 (0.60,304.72)	8.00 (1.66,38.45)	1.25 (0.37,4.29)	4.65 (0.91,23.81)	SUP

eTable 9C: Sensitive analyses: League Table for 9-month follow-up timepoint when limit to studies in which the population was in remission at the time of grouping

SUP+ADM								
7.38 (0.73,74.31)	CBT+ADM							
8.74 (0.89,85.75)	1.18 (0.64,2.19)	MBCT						
10.03 (0.87,115.90)	1.36 (0.46,3.98)	1.15 (0.41,3.17)	PLA					
10.22 (0.88,118.16)	1.39 (0.47,4.06)	1.17 (0.42,3.24)	1.02 (0.37,2.77)	IPT				
1.80 (1.26,2.58)	7.56 (0.97,58.94)	2.13 (1.29,3.52)	1.54 (0.59,3.99)	0.14 (0.02,0.81)	ADM			
114.25 (6.50,2009.53)	15.48 (2.46,97.52)	13.07 (2.14,79.67)	11.39 (1.53,85.06)	11.18 (1.50,83.49)	0.06 (0.01,0.46)	CBT		
189.61 (10.47,3434.86)	25.69 (3.90,169.40)	21.69 (3.40,138.50)	18.90 (2.43,147.20)	18.55 (2.38,144.47)	5.03 (1.27,19.92)	1.66 (1.10,2.51)	TAU	
913.81 (38.76,21545.75)	123.82 (12.79,1198.68)	104.54 (11.09,985.44)	91.11 (8.18,1014.53)	89.39 (8.03,995.68)	0.95 (0.30,3.02)	8.00 (2.12,30.22)	4.82 (1.20,19.38)	SUP

eTable 9D: Sensitive analyses: League Table for 12-month follow-up timepoint when limit to studies in which the population was in remission at the time of grouping

CBT+ADM								
1.15 (0.63,2.09)	SUP+ADM							
1.42 (0.81,2.50)	1.24 (0.54,2.82)	MBCT						
1.68 (0.66,4.26)	1.46 (0.48,4.42)	1.18 (0.49,2.86)	IPT					
1.95 (0.78,4.87)	1.70 (0.57,5.06)	1.37 (0.58,3.26)	1.16 (0.52,2.60)	PLA				
5.53 (0.98,31.30)	1.54 (1.09,2.18)	2.19 (1.40,3.44)	0.22 (0.05,0.88)	1.30 (0.58,2.94)	ADM			
10.20 (2.31,44.97)	8.87 (1.79,43.92)	7.17 (1.67,30.73)	6.06 (1.19,30.96)	5.23 (1.03,26.49)	0.07 (0.01,0.38)	CBT		
15.95 (3.44,73.96)	13.87 (2.67,71.98)	11.22 (2.49,50.59)	9.48 (1.77,50.70)	8.18 (1.54,43.39)	5.96 (1.62,21.97)	1.56 (1.06,2.31)	TAU	
72.65 (10.79,489.09)	63.18 (8.56,466.17)	51.10 (7.76,336.41)	43.17 (5.71,326.60)	37.26 (4.96,279.95)	1.13 (0.37,3.49)	7.12 (2.15,23.60)	4.56 (1.29,16.06)	SUP

eTable 9E: Sensitive analyses: League Table for 15-month follow-up timepoint when limit to studies in which the population was in remission at the time of grouping

CBT+ADM								
1.14 (0.64,2.06)	SUP+ADM							
1.30 (0.75,2.24)	1.14 (0.51,2.53)	MBCT						
1.49 (0.61,3.64)	1.30 (0.45,3.80)	1.15 (0.49,2.70)	IPT					
1.58 (0.65,3.83)	1.38 (0.48,4.00)	1.22 (0.52,2.84)	1.06 (0.48,2.32)	PLA				
0.46 (0.13,1.57)	0.47 (0.13,1.75)	1.29 (0.59,2.84)	1.93 (1.26,2.95)	0.92 (0.30,2.83)	ADM			
4.22 (1.14,15.57)	3.69 (0.88,15.44)	3.25 (0.90,11.69)	2.83 (0.66,12.22)	2.67 (0.62,11.49)	0.21 (0.05,0.94)	CBT		
6.51 (1.67,25.36)	5.69 (1.29,25.02)	5.01 (1.32,19.05)	4.36 (0.96,19.78)	4.12 (0.91,18.61)	0.82 (0.38,1.78)	1.54 (1.05,2.26)	TAU	
34.36 (6.18,190.95)	30.03 (4.90,184.04)	26.45 (4.85,144.22)	23.03 (3.67,144.71)	21.75 (3.47,136.20)	0.67 (0.48,0.95)	8.14 (2.67,24.77)	5.28 (1.63,17.13)	SUP

eTable 9F: Sensitive analyses: League Table for 18-month follow-up timepoint when limit to studies in which the population was in remission at the time of grouping

CBT+ADM								
1.09 (0.61,1.96)	SUP+ADM							
1.20 (0.51,2.82)	1.10 (0.39,3.09)	PLA						
1.23 (0.52,2.89)	1.13 (0.40,3.17)	1.02 (0.48,2.18)	IPT					
1.38 (0.79,2.41)	1.26 (0.56,2.84)	1.15 (0.50,2.66)	1.12 (0.48,2.60)	MBCT				
0.52 (0.16,1.76)	0.33 (0.09,1.20)	1.03 (0.33,3.21)	1.84 (1.22,2.76)	1.49 (0.70,3.16)	ADM			
3.51 (0.98,12.62)	3.21 (0.79,13.11)	2.92 (0.70,12.15)	2.85 (0.69,11.87)	2.54 (0.71,9.07)	0.40 (0.10,1.65)	CBT		
5.10 (1.34,19.37)	4.67 (1.09,20.03)	4.24 (0.97,18.54)	4.14 (0.95,18.11)	3.69 (0.98,13.92)	0.65 (0.31,1.38)	1.45 (0.99,2.12)	TAU	
38.22 (6.99,209.07)	34.97 (5.80,210.92)	31.79 (5.19,194.55)	31.03 (5.07,190.03)	27.68 (5.09,150.53)	0.75 (0.51,1.10)	10.88 (3.55,33.31)	7.49 (2.30,24.41)	SUP

eTable 10A: Sensitive analyses: League Table for 3-month follow-up timepoint when limit to studies in which population was not the first episode of depression

MBCT+ADM							
3.01 (0.31,29.00)	MBCT						
3.79 (0.39,37.16)	1.26 (0.57,2.78)	CBT+ADM					
4.79 (0.43,53.43)	1.59 (0.52,4.83)	1.26 (0.58,2.75)	SUP+ADM				
1.77 (1.05,2.99)	0.51 (0.13,2.05)	1.41 (0.78,2.56)	3.03 (0.57,16.12)	ADM			
12.78 (1.03,158.53)	4.24 (1.13,15.97)	3.37 (0.87,13.10)	2.67 (0.56,12.74)	2.40 (0.71,8.10)	CBT		
25.38 (2.09,307.88)	8.43 (2.34,30.39)	6.70 (1.80,24.94)	5.30 (1.15,24.39)	0.19 (0.02,1.70)	1.99 (0.79,4.97)	PLA	
28.93 (2.20,380.39)	9.61 (2.29,40.26)	7.64 (1.77,32.93)	6.04 (1.15,31.62)	4.76 (1.48,15.35)	2.26 (1.31,3.90)	1.14 (0.39,3.31)	TAU

eTable 10B: Sensitive analyses: League Table for 6-month follow-up timepoint when limit to studies in which population was not the first episode of depression

MBCT							
1.29 (0.67,2.45)	CBT+ADM						
1.35 (0.36,5.08)	1.05 (0.27,4.07)	MBCT+ADM					
1.60 (0.64,3.98)	1.24 (0.65,2.37)	1.18 (0.26,5.32)	SUP+ADM				
0.80 (0.23,2.73)	1.47 (0.89,2.42)	1.89 (1.26,2.84)	2.41 (0.62,9.36)	ADM			
3.03 (1.17,7.87)	2.36 (0.87,6.39)	2.25 (0.49,10.36)	1.90 (0.58,6.23)	1.61 (0.68,3.81)	CBT		
5.21 (1.81,14.96)	4.05 (1.36,12.10)	3.86 (0.79,19.01)	3.26 (0.92,11.62)	2.89 (1.24,6.72)	1.72 (1.10,2.69)	TAU	
5.45 (2.13,13.93)	4.24 (1.59,11.32)	4.04 (0.89,18.45)	3.41 (1.05,11.06)	0.71 (0.20,2.52)	1.80 (0.83,3.88)	1.05 (0.43,2.55)	PLA

eTable 10C: Sensitive analyses: League Table for 9-month follow-up timepoint when limit to studies in which population was not the first episode of depression

CBT+ADM							
1.15 (0.55,2.41)	MBCT						
1.16 (0.63,2.15)	1.01 (0.38,2.63)	SUP+ADM					
1.66 (0.47,5.85)	1.44 (0.46,4.51)	1.43 (0.35,5.82)	MBCT+ADM				
2.08 (0.76,5.66)	1.80 (0.77,4.21)	1.79 (0.55,5.81)	1.25 (0.33,4.71)	CBT			
2.08 (1.09,3.95)	1.05 (0.33,3.32)	2.19 (0.62,7.77)	1.80 (1.26,2.58)	1.00 (0.46,2.16)	ADM		
3.45 (1.17,10.18)	2.99 (1.16,7.68)	2.97 (0.85,10.34)	2.07 (0.52,8.33)	1.66 (1.10,2.51)	2.19 (1.05,4.58)	TAU	
4.55 (1.71,12.10)	3.94 (1.73,8.96)	3.92 (1.23,12.46)	2.73 (0.74,10.14)	2.19 (1.05,4.58)	0.80 (0.27,2.36)	1.32 (0.57,3.07)	PLA

eTable 10D: Sensitive analyses: League Table for 12-month follow-up timepoint when limit to studies in which population was not the first episode of depression

CBT+ADM							
1.15 (0.63,2.09)	SUP+ADM						
1.49 (0.58,3.82)	1.30 (0.43,3.95)	CBT					
1.52 (0.75,3.07)	1.32 (0.53,3.33)	1.02 (0.46,2.24)	MBCT				
1.76 (0.55,5.64)	1.53 (0.41,5.67)	1.18 (0.35,3.99)	1.16 (0.41,3.30)	MBCT+ADM			
2.34 (0.85,6.45)	2.03 (0.62,6.60)	1.56 (1.06,2.31)	1.54 (0.64,3.70)	1.33 (0.37,4.77)	TAU		
2.35 (1.27,4.33)	1.29 (0.37,4.46)	1.57 (0.77,3.19)	0.88 (0.29,2.73)	1.54 (1.09,2.18)	0.77 (0.39,1.54)	ADM	
3.03 (1.21,7.62)	2.64 (0.88,7.91)	2.03 (0.98,4.21)	1.99 (0.92,4.30)	1.72 (0.52,5.75)	1.30 (0.57,2.97)	0.75 (0.28,2.02)	PLA

eTable 10E: Sensitive analyses: League Table for 15-month follow-up timepoint when limit to studies in which population was not the first episode of depression

CBT+ADM							
1.14 (0.64,2.06)	SUP+ADM						
1.28 (0.65,2.54)	1.12 (0.46,2.76)	MBCT					
1.35 (0.55,3.30)	1.18 (0.40,3.43)	1.05 (0.49,2.23)	CBT				
1.65 (0.53,5.14)	1.45 (0.40,5.19)	1.29 (0.46,3.59)	1.23 (0.38,3.98)	MBCT+ADM			
1.91 (1.05,3.45)	0.67 (0.19,2.35)	0.92 (0.30,2.83)	1.42 (0.72,2.77)	1.49 (1.06,2.09)	ADM		
2.08 (0.78,5.50)	1.81 (0.58,5.66)	1.62 (0.70,3.76)	1.54 (1.05,2.26)	1.26 (0.36,4.33)	1.17 (0.59,2.28)	TAU	
2.22 (0.91,5.45)	1.94 (0.67,5.68)	1.73 (0.81,3.68)	1.65 (0.82,3.32)	1.34 (0.41,4.37)	0.87 (0.33,2.28)	1.07 (0.48,2.37)	PLA

eTable 10F: Sensitive analyses: League Table for 18-month follow-up timepoint when limit to studies in which population was not the first episode of depression

CBT+ADM						
1.09 (0.61,1.96)	SUP+ADM					
1.32 (0.66,2.67)	1.21 (0.49,3.01)	MBCT				
1.50 (0.63,3.57)	1.37 (0.48,3.91)	1.13 (0.54,2.39)	CBT			
1.85 (0.76,4.49)	1.69 (0.58,4.89)	1.39 (0.65,3.01)	1.23 (0.63,2.42)	PLA		
1.76 (0.98,3.16)	0.52 (0.15,1.84)	1.03 (0.33,3.21)	1.17 (0.62,2.23)	1.33 (0.91,1.95)	ADM	
2.18 (0.84,5.62)	1.99 (0.65,6.07)	1.65 (0.71,3.80)	1.45 (0.99,2.12)	1.18 (0.54,2.56)	1.05 (0.54,2.04)	TAU

eTable 11A: Sensitive analyses: League Table for 3-month follow-up timepoint when exclude arms that included placebo

SUP+ADM										
2.86 (0.07,119.46)	MBCT+ADM									
9.47 (0.45,199.96)	3.31 (0.35,31.61)	MBCT								
12.72 (0.46,353.35)	4.44 (0.32,60.79)	1.34 (0.30,5.96)	CBT+ADM							
13.93 (0.54,356.71)	4.87 (0.40,59.98)	1.47 (0.40,5.38)	1.10 (0.17,6.99)	CBT						
14.21 (0.46,438.78)	4.97 (0.32,77.58)	1.50 (0.27,8.31)	1.12 (0.13,9.73)	1.02 (0.13,7.81)	IPT					
16.02 (0.62,411.82)	5.60 (0.45,69.32)	1.69 (0.46,6.25)	1.26 (0.20,8.09)	1.15 (0.34,3.84)	1.13 (0.15,8.69)	BA				
1.07 (0.21,5.55)	1.51 (0.34,6.78)	1.10 (0.33,3.65)	4.66 (0.54,39.93)	0.83 (0.19,3.69)	2.94 (0.22,39.20)	0.95 (0.28,3.21)	ADM			
31.55 (1.18,845.08)	11.02 (0.84,143.94)	3.33 (0.82,13.59)	2.48 (0.36,17.10)	2.26 (1.31,3.90)	2.22 (0.27,18.27)	1.97 (0.52,7.40)	0.62 (0.38,1.01)	TAU		
75.99 (2.04,2825.71)	26.56 (1.35,521.50)	8.02 (1.02,62.91)	5.98 (0.52,69.09)	5.45 (1.10,27.01)	5.35 (0.40,71.25)	4.74 (0.64,35.19)	0.83 (0.20,3.41)	2.41 (0.44,13.05)	SUP	

eTable 11B: Sensitive analyses: League Table for 6-month follow-up timepoint when exclude arms that included placebo

SUP+ADM									
8.87 (0.85,92.87)	MBCT								
10.13 (0.67,153.34)	1.14 (0.25,5.31)	CBT+ADM							
11.25 (0.79,159.79)	1.27 (0.31,5.25)	1.11 (0.15,7.99)	MBCT+ADM						
11.94 (0.89,159.76)	1.35 (0.36,4.97)	1.18 (0.18,7.82)	1.06 (0.18,6.41)	BA					
14.17 (1.08,185.85)	1.60 (0.45,5.67)	1.40 (0.22,9.03)	1.26 (0.21,7.39)	1.19 (0.36,3.94)	CBT				
14.58 (1.10,193.90)	1.64 (0.45,6.00)	1.44 (0.22,9.47)	1.30 (0.22,7.76)	1.22 (0.22,6.69)	1.03 (0.19,5.46)	IPT			
1.08 (0.33,3.57)	1.11 (0.35,3.56)	2.62 (0.38,18.11)	1.24 (0.27,5.76)	1.32 (0.39,4.42)	0.94 (0.24,3.73)	1.36 (0.24,7.70)	ADM		
24.36 (1.76,337.94)	2.75 (0.69,10.88)	2.41 (0.35,16.76)	2.17 (0.34,13.78)	2.04 (0.55,7.62)	1.72 (1.00,2.95)	1.67 (0.29,9.66)	0.56 (0.34,0.93)	TAU	
113.38 (5.40,2381.50)	12.78 (1.63,100.42)	11.20 (0.94,132.96)	10.08 (0.91,111.51)	9.50 (1.26,71.75)	8.00 (1.57,40.71)	7.77 (0.76,79.96)	0.64 (0.15,2.76)	4.65 (0.84,25.86)	SUP

eTable 11C: Sensitive analyses: League Table for 9-month follow-up timepoint when exclude arms that included placebo

SUP+ADM									
8.70 (0.89,85.12)	MBCT								
7.80 (0.56,108.99)	0.90 (0.22,3.67)	CBT+ADM							
10.22 (0.88,118.16)	1.17 (0.43,3.23)	1.31 (0.25,6.95)	IPT						
12.17 (1.00,147.81)	1.40 (0.45,4.31)	1.56 (0.27,8.89)	1.19 (0.28,5.00)	CBT					
12.60 (1.03,153.67)	1.45 (0.47,4.51)	1.62 (0.28,9.25)	1.23 (0.29,5.21)	1.04 (0.23,4.75)	MBCT+ADM				
14.32 (1.17,175.09)	1.65 (0.53,5.15)	1.84 (0.32,10.56)	1.40 (0.33,5.95)	1.18 (0.40,3.50)	1.14 (0.24,5.28)	BA			
0.36 (0.06,2.24)	1.29 (0.44,3.78)	3.73 (0.60,23.19)	1.82 (0.40,8.21)	0.63 (0.19,2.14)	1.10 (0.26,4.61)	1.10 (0.37,3.27)	ADM		
20.20 (1.61,253.75)	2.32 (0.70,7.70)	2.59 (0.43,15.48)	1.98 (0.44,8.79)	1.66 (1.10,2.51)	1.60 (0.33,7.77)	1.41 (0.44,4.52)	0.65 (0.25,1.68)	TAU	
66.90 (4.71,950.43)	7.69 (1.82,32.47)	8.58 (1.21,60.77)	6.54 (1.20,35.56)	5.50 (2.24,13.50)	5.31 (0.91,31.15)	4.67 (1.14,19.18)	0.49 (0.13,1.95)	3.31 (1.23,8.90)	SUP

eTable 11D: Sensitive analyses: League Table for 12-month follow-up timepoint when exclude arms that included placebo

CBT+ADM									
1.15 (0.63,2.09)	SUP+ADM								
1.59 (0.27,9.25)	1.38 (0.21,8.88)	CBT							
1.80 (0.42,7.65)	1.57 (0.33,7.49)	1.14 (0.37,3.45)	MBCT						
2.06 (0.37,11.53)	1.79 (0.29,11.09)	1.30 (0.30,5.55)	1.14 (0.40,3.25)	MBCT+ADM					
2.11 (0.42,10.72)	1.83 (0.32,10.37)	1.33 (0.35,5.07)	1.17 (0.49,2.81)	1.02 (0.28,3.69)	IPT				
2.54 (0.44,14.80)	2.21 (0.34,14.21)	1.60 (0.55,4.68)	1.41 (0.46,4.28)	1.23 (0.29,5.26)	1.20 (0.32,4.58)	BA			
2.48 (0.41,15.10)	2.16 (0.32,14.46)	1.56 (1.06,2.31)	1.38 (0.42,4.47)	1.20 (0.27,5.41)	1.18 (0.29,4.74)	0.98 (0.31,3.06)	TAU		
4.76 (0.74,30.59)	0.57 (0.12,2.74)	0.63 (0.19,2.07)	1.73 (0.60,5.01)	0.78 (0.18,3.34)	1.82 (0.43,7.68)	1.08 (0.37,3.13)	1.30 (0.58,2.94)	ADM	
7.14 (1.03,49.48)	6.21 (0.82,47.10)	4.50 (2.03,10.00)	3.96 (1.01,15.56)	3.46 (0.66,18.16)	3.39 (0.71,16.07)	2.81 (0.74,10.72)	2.88 (1.18,7.00)	0.36 (0.09,1.49)	SUP

eTable 11E: Sensitive analyses: League Table for 15-month follow-up timepoint when exclude arms that included placebo

CBT									
1.20 (0.42,3.44)	BA								
1.54 (1.05,2.26)	1.28 (0.42,3.94)	TAU							
1.99 (0.63,6.35)	1.66 (0.51,5.39)	1.29 (0.38,4.38)	CBT+ADM						
2.28 (0.62,8.37)	1.90 (0.51,7.09)	1.48 (0.38,5.74)	1.14 (0.64,2.06)	SUP+ADM					
2.54 (0.81,7.99)	2.12 (0.66,6.78)	1.65 (0.49,5.52)	1.27 (0.78,2.09)	1.11 (0.52,2.40)	MBCT				
2.90 (0.75,11.17)	2.41 (0.62,9.46)	1.88 (0.46,7.64)	1.45 (0.61,3.46)	1.27 (0.44,3.62)	1.14 (0.49,2.66)	IPT			
3.25 (0.75,14.04)	2.71 (0.62,11.87)	2.11 (0.46,9.57)	1.63 (0.58,4.59)	1.42 (0.43,4.68)	1.28 (0.46,3.54)	1.12 (0.32,3.90)	MBCT+ADM		
3.81 (1.86,7.80)	3.18 (0.89,11.36)	2.47 (1.10,5.57)	1.91 (0.49,7.47)	1.67 (0.38,7.37)	1.50 (0.39,5.79)	1.32 (0.29,6.07)	1.17 (0.23,5.99)	SUP	
0.29 (0.08,1.02)	3.12 (1.02,9.55)	1.15 (0.44,3.04)	0.58 (0.14,2.34)	1.47 (1.07,2.04)	1.88 (1.30,2.72)	3.75 (1.25,11.24)	1.41 (0.29,6.75)	1.29 (0.59,2.84)	ADM

eTable 11F: Sensitive analyses: League Table for 18-month follow-up timepoint when exclude arms that included placebo

CBT								
1.21 (0.43,3.45)	BA							
1.45 (0.99,2.12)	1.20 (0.39,3.64)	TAU						
2.22 (0.69,7.18)	1.83 (0.55,6.04)	1.53 (0.45,5.25)	CBT+ADM					
2.43 (0.65,9.01)	2.00 (0.53,7.56)	1.67 (0.43,6.54)	1.09 (0.61,1.96)	SUP+ADM				
2.63 (0.68,10.12)	2.17 (0.55,8.48)	1.81 (0.45,7.34)	1.18 (0.52,2.71)	1.08 (0.39,2.99)	IPT			
2.94 (0.91,9.53)	2.42 (0.73,8.01)	2.02 (0.59,6.96)	1.32 (0.80,2.20)	1.21 (0.56,2.62)	1.12 (0.49,2.57)	MBCT		
3.59 (1.78,7.27)	2.96 (0.84,10.43)	2.47 (1.11,5.50)	1.62 (0.41,6.36)	1.48 (0.33,6.56)	1.37 (0.30,6.25)	1.22 (0.31,4.81)	SUP	
0.25 (0.07,0.88)	3.23 (1.03,10.11)	1.33 (0.93,1.91)	0.34 (0.09,1.29)	1.49 (0.70,3.16)	3.92 (1.28,12.01)	1.77 (1.24,2.52)	1.43 (0.30,6.74)	ADM

eTable 11G: Sensitive analyses: League Table for 21-month follow-up timepoint when exclude arms that included placebo

CBT							
1.43 (0.98,2.08)	TAU						
1.55 (0.74,3.25)	1.09 (0.47,2.50)	IPT					
1.63 (0.57,4.66)	1.15 (0.38,3.49)	1.05 (0.29,3.80)	BA				
3.07 (1.53,6.14)	2.15 (0.98,4.74)	1.98 (0.72,5.45)	1.88 (0.53,6.60)	SUP			
4.70 (1.25,17.72)	3.29 (0.83,13.09)	3.03 (0.66,13.84)	2.87 (0.75,11.07)	1.53 (0.34,6.85)	CBT+ADM		
6.01 (1.58,22.90)	4.22 (1.05,16.92)	3.88 (0.84,17.86)	3.68 (0.95,14.30)	1.96 (0.43,8.84)	1.28 (0.76,2.15)	MBCT	
0.10 (0.02,0.39)	0.38 (0.07,2.16)	1.67 (1.18,2.36)	4.80 (1.30,17.64)	1.30 (0.89,1.91)	7.84 (2.18,28.22)	5.05 (1.15,22.18)	ADM

eTable 11H: Sensitive analyses: League Table for 24-month follow-up timepoint when exclude arms that included placebo

CBT							
1.34 (0.82,2.19)	TAU						
1.43 (0.46,4.42)	1.06 (0.31,3.65)	BA					
1.43 (0.62,3.33)	1.07 (0.40,2.84)	1.00 (0.25,4.11)	IPT				
3.71 (0.90,15.25)	2.77 (0.62,12.37)	2.60 (0.62,10.91)	2.59 (0.50,13.41)	CBT+ADM			
3.09 (1.35,7.08)	2.30 (0.89,5.97)	2.16 (0.53,8.79)	2.16 (0.66,7.03)	0.83 (0.16,4.32)	SUP		
5.57 (1.28,24.13)	4.15 (0.88,19.49)	3.90 (0.88,17.25)	3.88 (0.71,21.06)	1.50 (0.74,3.05)	1.80 (0.33,9.71)	MBCT	
0.13 (0.03,0.62)	0.41 (0.06,2.88)	4.80 (1.22,18.91)	1.84 (1.21,2.80)	6.85 (1.78,26.40)	1.23 (0.69,2.19)	4.78 (0.97,23.44)	ADM

eCINeMA

A Summary of within-study bias of the included studies

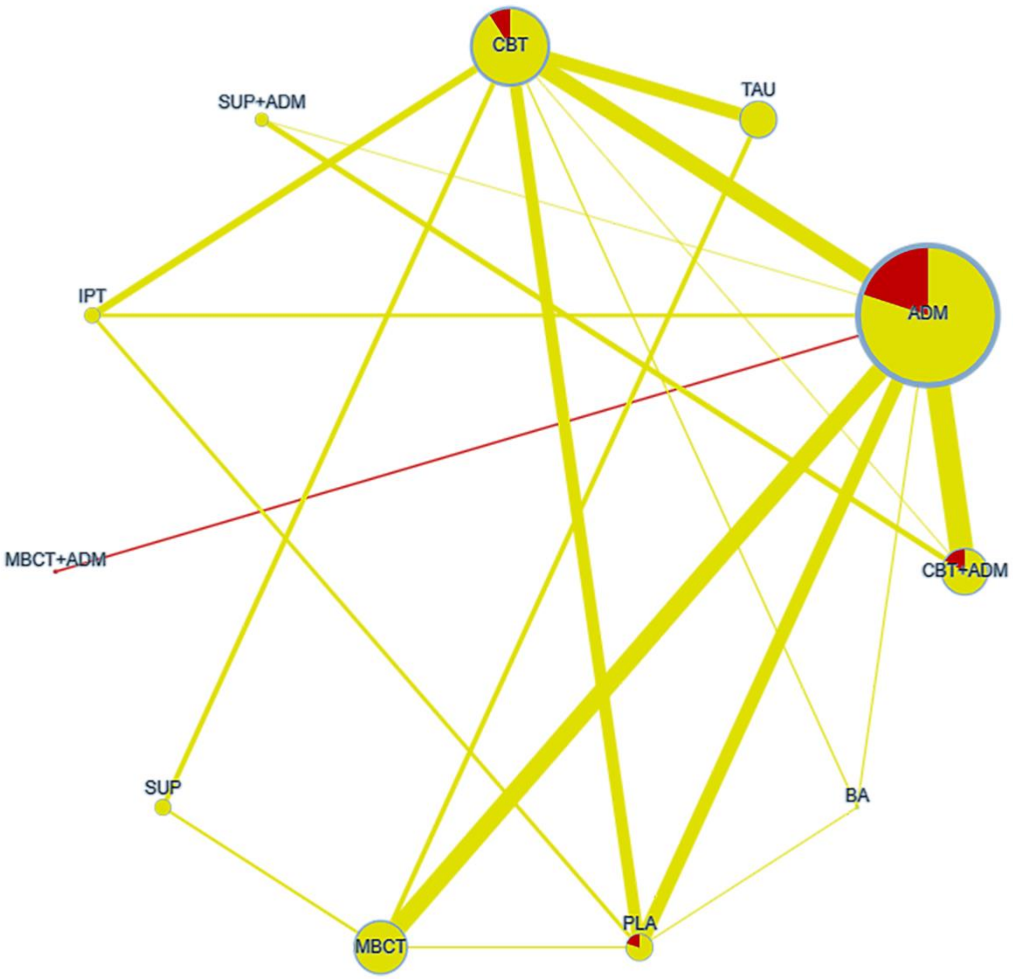

B Contribution of comparisons to each network estimate by Overall RoB

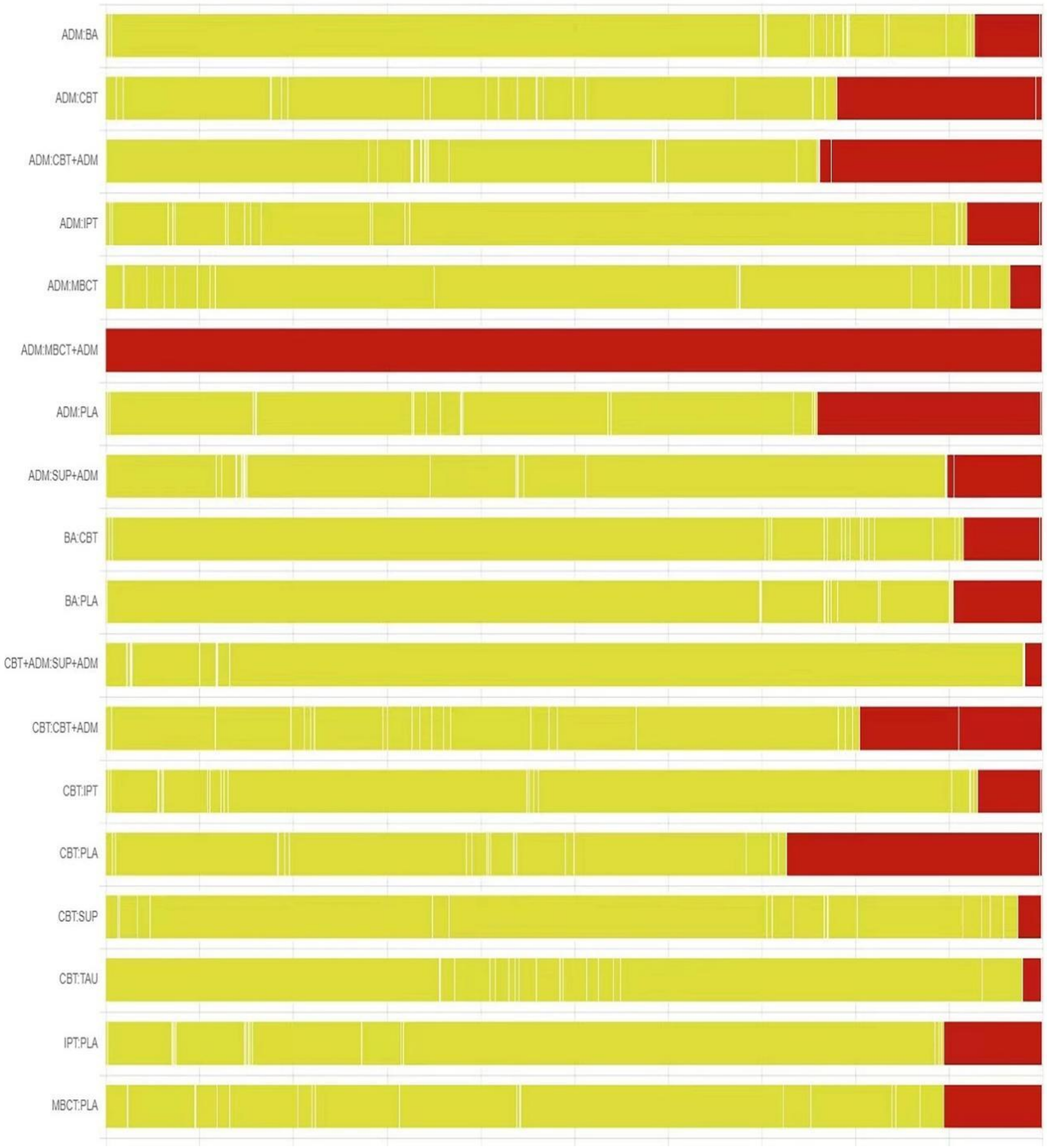

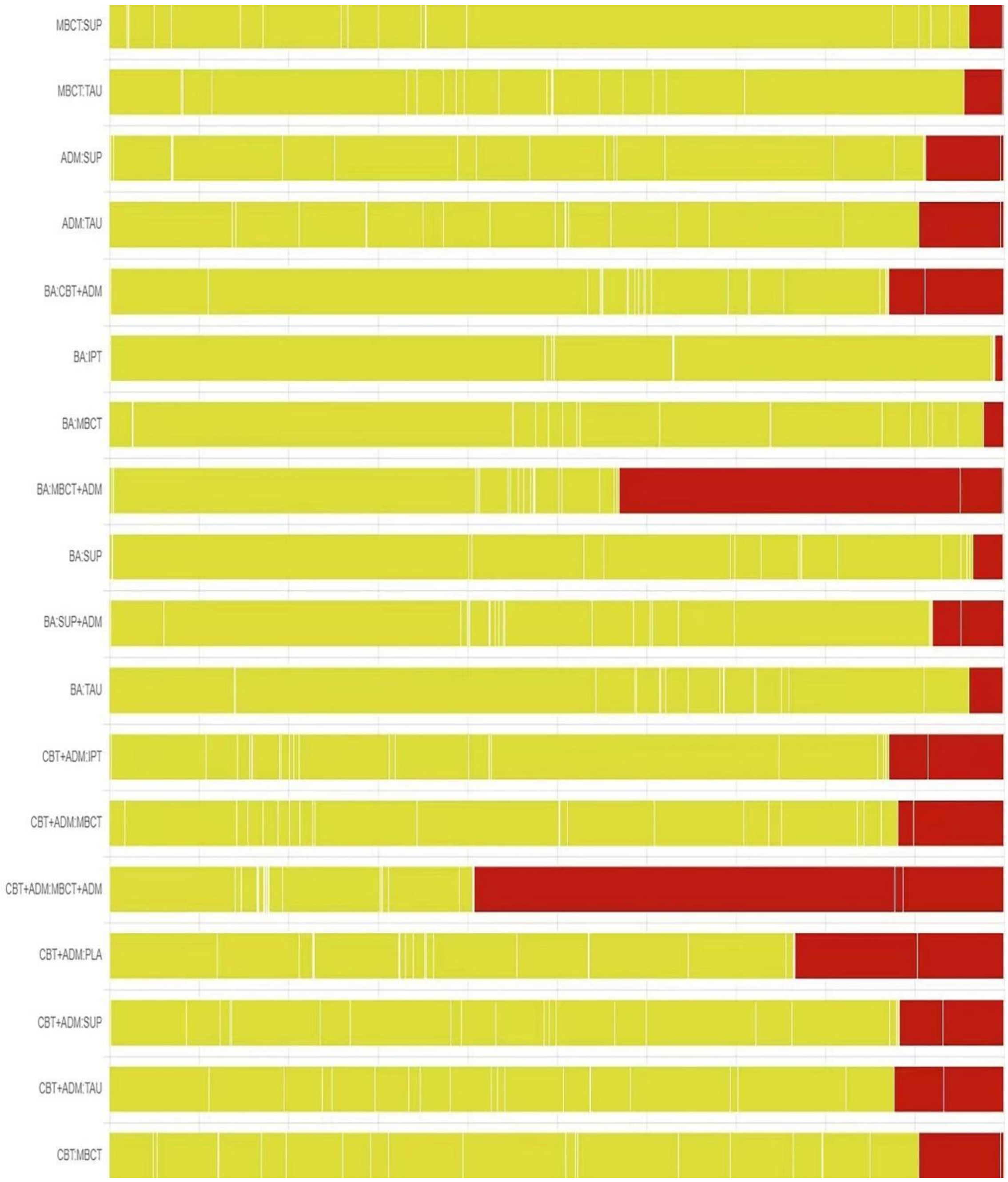
MBCT:SUP
MBCT:TAU
ADM:SUP
ADM:TAU
BA:CBT+ADM
BA:IPT
BA:MBCT
BA:MBCT+ADM
BA:SUP
BA:SUP+ADM
BA:TAU
CBT+ADM:IPT
CBT+ADM:MBCT
CBT+ADM:MBCT+ADM
CBT+ADM:PLA
CBT+ADM:SUP
CBT+ADM:TAU
CBT:MBCT

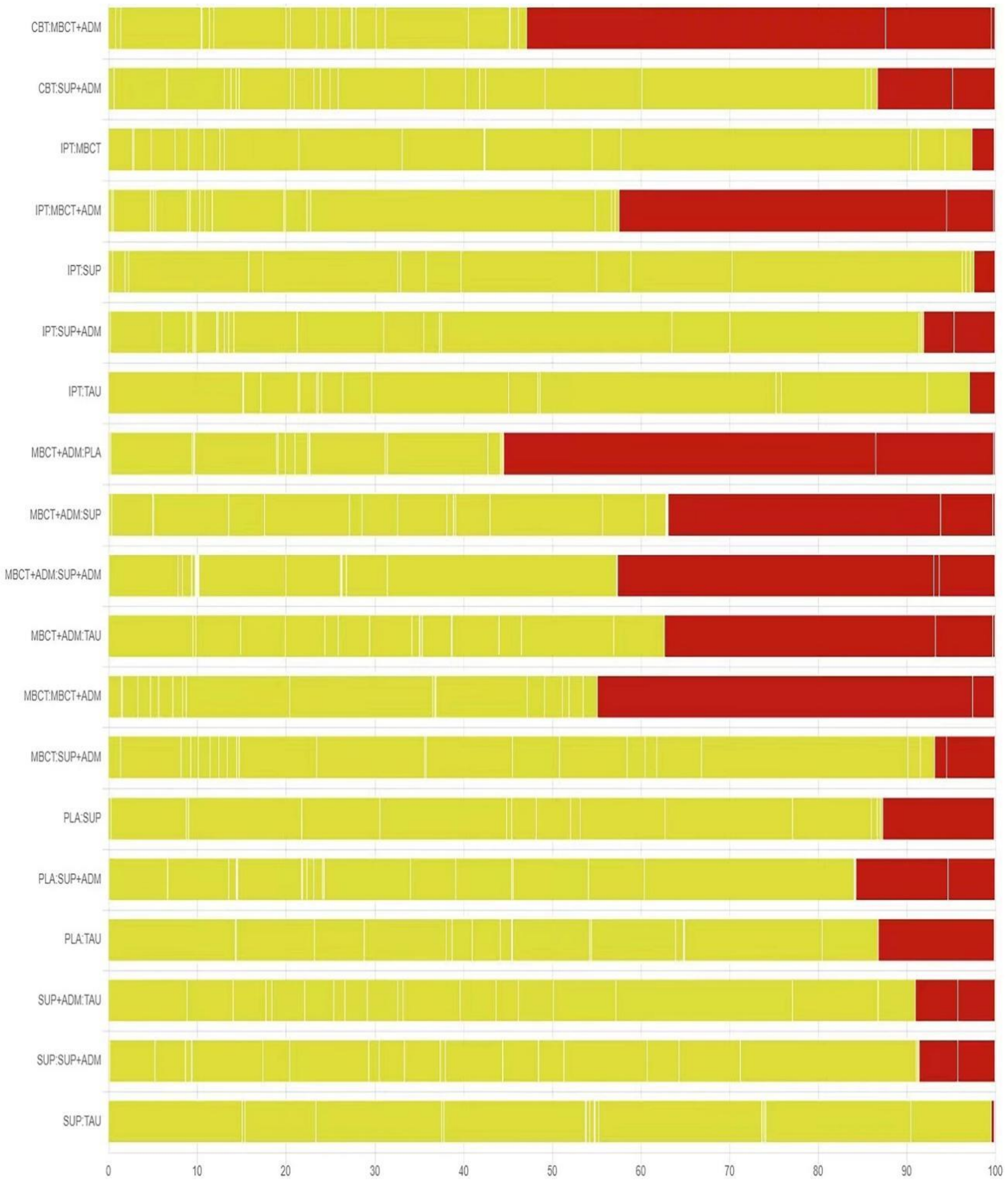
CBT:MBCT+ADM
CBT:SUP+ADM
IPT:MBCT
IPT:MBCT+ADM
IPT:SUP
IPT:SUP+ADM
IPT:TAU
MBCT+ADM:PLA
MBCT+ADM:SUP
MBCT+ADM:SUP+ADM
MBCT+ADM:TAU
MBCT:MBCT+ADM
MBCT:SUP+ADM
PLA:SUP
PLA:SUP+ADM
PLA:TAU
SUP+ADM:TAU
SUP:SUP+ADM
SUP:TAU
0
10
20
30
40
50
60
70
80
90
100

C Confidence in evidence

Comparison	Number of studies	Within-study bias	Reporting bias	Indirectness	Imprecision	Heterogeneity	Incoherence	Confidence rating
ADM:BA	1	Some concerns	Low risk	No concerns	Major concerns	No concerns	No concerns	Low
ADM:CBT	6	Some concerns	Low risk	No concerns	Major concerns	No concerns	No concerns	Low
ADM:CBT+ADM	5	Some concerns	Low risk	No concerns	No concerns	Major concerns	No concerns	Low
ADM:IPT	1	Some concerns	Low risk	No concerns	Major concerns	No concerns	No concerns	Low
ADM:MBCT	3	Some concerns	Low risk	No concerns	No concerns	Major concerns	No concerns	Low
ADM:MBCT+ADM	1	Major concerns	Low risk	No concerns	Major concerns	No concerns	Major concerns	Very low
ADM:PLA	5	Some concerns	Low risk	No concerns	Major concerns	No concerns	No concerns	Low
ADM:SUP+ADM	1	Some concerns	Low risk	No concerns	Major concerns	No concerns	No concerns	Low
BA:CBT	1	Some concerns	Low risk	No concerns	Major concerns	No concerns	No concerns	Low
BA:PLA	1	Some concerns	Low risk	No concerns	Major concerns	No concerns	No concerns	Low
CBT:CBT+ADM	1	Some concerns	Low risk	No concerns	Major concerns	No concerns	No concerns	Low
CBT:IPT	2	Some concerns	Low risk	No concerns	Major concerns	No concerns	No concerns	Low
CBT:PLA	4	Some concerns	Low risk	No concerns	No concerns	Major concerns	No concerns	Low
CBT:SUP	2	Some concerns	Low risk	No concerns	No concerns	Major concerns	Major concerns	Low
CBT:TAU	2	Some concerns	Low risk	No concerns	No concerns	Major concerns	Major concerns	Low
CBT+ADM:SUP+ADM	1	Some concerns	Low risk	No concerns	Major concerns	No concerns	No concerns	Low
IPT:PLA	1	Some concerns	Low risk	No concerns	Major concerns	No concerns	No concerns	Low
MBCT:PLA	1	Some concerns	Low risk	No concerns	No concerns	Major concerns	No concerns	Low
MBCT:SUP	1	Some concerns	Low risk	No concerns	No concerns	Major concerns	Major concerns	Low
MBCT:TAU	2	Some concerns	Low risk	No concerns	No concerns	No concerns	Major concerns	Low
ADM:SUP	0	Some concerns	Low risk	No concerns	Major concerns	No concerns	Major concerns	Very low
ADM:TAU	0	Some concerns	Low risk	No concerns	Major concerns	No concerns	Major concerns	Very low

BA:CBT+ADM	0	Some concerns	Low risk	No concerns	Major concerns	No concerns	Major concerns	Very low
BA:IPT	0	Some concerns	Low risk	No concerns	Major concerns	No concerns	Major concerns	Very low
BA:MBCT	0	Some concerns	Low risk	No concerns	Major concerns	No concerns	Major concerns	Very low
BA:MBCT+ADM	0	Some concerns	Low risk	No concerns	Major concerns	No concerns	Major concerns	Very low
BA:SUP	0	Some concerns	Low risk	No concerns	Major concerns	No concerns	Major concerns	Very low
BA:SUP+ADM	0	Some concerns	Low risk	No concerns	Major concerns	No concerns	Major concerns	Very low
BA:TAU	0	Some concerns	Low risk	No concerns	Major concerns	No concerns	Major concerns	Very low
CBT:MBCT	0	Some concerns	Low risk	No concerns	Major concerns	No concerns	Major concerns	Very low
CBT:MBCT+ADM	0	Major concerns	Low risk	No concerns	Major concerns	No concerns	Major concerns	Very low
CBT:SUP+ADM	0	Some concerns	Low risk	No concerns	Major concerns	No concerns	Major concerns	Very low
CBT+ADM:IPT	0	Some concerns	Low risk	No concerns	Major concerns	No concerns	Major concerns	Very low
CBT+ADM:MBCT	0	Some concerns	Low risk	No concerns	Major concerns	No concerns	Major concerns	Very low
CBT+ADM:MBCT+ADM	0	Major concerns	Low risk	No concerns	Major concerns	No concerns	Major concerns	Very low
CBT+ADM:PLA	0	Some concerns	Low risk	No concerns	No concerns	No concerns	Major concerns	Low
CBT+ADM:SUP	0	Some concerns	Low risk	No concerns	No concerns	No concerns	Major concerns	Low
CBT+ADM:TAU	0	Some concerns	Low risk	No concerns	No concerns	No concerns	Major concerns	Low
IPT:MBCT	0	Some concerns	Low risk	No concerns	Major concerns	No concerns	Major concerns	Very low
IPT:MBCT+ADM	0	Some concerns	Low risk	No concerns	Major concerns	No concerns	Major concerns	Very low
IPT:SUP	0	Some concerns	Low risk	No concerns	Major concerns	No concerns	Major concerns	Very low
IPT:SUP+ADM	0	Some concerns	Low risk	No concerns	Major concerns	No concerns	Major concerns	Very low
IPT:TAU	0	Some concerns	Low risk	No concerns	Major concerns	No concerns	Major concerns	Very low
MBCT:MBCT+ADM	0	Some concerns	Low risk	No concerns	Major concerns	No concerns	Major concerns	Very low
MBCT:SUP+ADM	0	Some concerns	Low risk	No concerns	Major concerns	No concerns	Major concerns	Very low
MBCT+ADM:PLA	0	Major concerns	Low risk	No concerns	Major concerns	No concerns	Major concerns	Very low

MBCT+ADM:SUP	0	Some concerns	Low risk	No concerns	Major concerns	No concerns	Major concerns	Very low
MBCT+ADM:SUP+ADM	0	Some concerns	Low risk	No concerns	Major concerns	No concerns	Major concerns	Very low
MBCT+ADM:TAU	0	Some concerns	Low risk	No concerns	Major concerns	No concerns	Major concerns	Very low
PLA:SUP	0	Some concerns	Low risk	No concerns	Major concerns	No concerns	Major concerns	Very low
PLA:SUP+ADM	0	Some concerns	Low risk	No concerns	No concerns	Major concerns	Major concerns	Low
PLA:TAU	0	Some concerns	Low risk	No concerns	Major concerns	No concerns	Major concerns	Very low
SUP:SUP+ADM	0	Some concerns	Low risk	No concerns	No concerns	Major concerns	Major concerns	Low
SUP:TAU	0	Some concerns	Low risk	No concerns	Major concerns	No concerns	Major concerns	Very low
SUP+ADM:TAU	0	Some concerns	Low risk	No concerns	No concerns	Major concerns	Major concerns	Low

ADM=antidepressant. BA=behavioural active therapy. CBT=cognitive behavioral therapy. CBT+ADM= cognitive behavioral therapy combined with antidepressant. IPT=interpersonal therapy. MBCT=mindfulness-based cognitive therapy. MBCT+ADM= mindfulness-based cognitive therapy combined with antidepressant. PLA= placebo. SUP=supportive counselling. SUP+ADM= supportive counselling combined with antidepressant. TAU=treatment as usual.
